# Modelling the immunological response to a tetravalent dengue vaccine from multiple phase-2 trials in Latin America and South East Asia

**DOI:** 10.1016/j.vaccine.2015.05.059

**Published:** 2015-07-17

**Authors:** Ilaria Dorigatti, Ricardo Aguas, Christl A. Donnelly, Bruno Guy, Laurent Coudeville, Nicholas Jackson, Melanie Saville, Neil M. Ferguson

**Affiliations:** a MRC Centre for Outbreak Analysis and Modelling, Department of Infectious Disease Epidemiology, School of Public Health, Imperial College London, Norfolk Place, W2 1PG London, United Kingdom; b Sanofi Pasteur, 2 Avenue Pont Pasteur, Lyon Cedex 07 69367, France

**Keywords:** CYD-TDV dengue vaccine, PRNT50 antibody titres, Multivariate regression, Serotype interactions

## Abstract

**Background:**

The most advanced dengue vaccine candidate is a live-attenuated recombinant vaccine containing the four dengue viruses on the yellow fever vaccine backbone (CYD-TDV) developed by Sanofi Pasteur. Several analyses have been published on the safety and immunogenicity of the CYD-TDV vaccine from single trials but none modelled the heterogeneity observed in the antibody responses elicited by the vaccine.

**Methods:**

We analyse the immunogenicity data collected in five phase-2 trials of the CYD-TDV vaccine. We provide a descriptive analysis of the aggregated datasets and fit the observed post-vaccination PRNT50 titres against the four dengue (DENV) serotypes using multivariate regression models.

**Results:**

We find that the responses to CYD-TDV are principally predicted by the baseline immunological status against DENV, but the trial is also a significant predictor. We find that the CYD-TDV vaccine generates similar titres against all serotypes following the third dose, though DENV4 is immunodominant after the first dose.

**Conclusions:**

This study contributes to a better understanding of the immunological responses elicited by CYD-TDV. The recent availability of phase-3 data is a unique opportunity to further investigate the immunogenicity and efficacy of the CYD-TDV vaccine, especially in subjects with different levels of pre-existing immunity against DENV.

Modelling multiple immunological outcomes with a single multivariate model offers advantages over traditional approaches, capturing correlations between response variables, and the statistical method adopted in this study can be applied to a variety of infections with interacting strains.

## 1 Introduction

Dengue is a systemic vector-borne viral infection caused by four distinct virus serotypes (DENV1–4) which are transmitted by *Aedes* mosquitoes. Recent estimates suggest that worldwide, dengue infects 390 million people annually [1] and that more than half of the world's population is at risk of dengue infection [2]. Currently, there is no antiviral treatment, so future hopes for control rely on the development of an effective dengue vaccine [2,3] and on improved vector control [4,5]. The most advanced dengue vaccine candidate (CYD-TDV) is a recombinant, live-attenuated, tetravalent vaccine constructed on the Yellow Fever Vaccine (YFV) 17D backbone. Several descriptive analyses have been published on the safety and immunogenicity of CYD-TDV using data from single trials [6–12] but none of these studies modelled the heterogeneity observed in the antibody responses elicited by the vaccine. Moreover, the first phase-2b efficacy trial [9] suggested that efficacy varied by serotype, with no statistically significant efficacy against DENV2 observed.

In this work we analyse the immunogenicity data collected in five phase-2, randomised, observer-blind, controlled trials of the CYD-TDV dengue vaccine [6,9–12]. Using multivariate regression models we identify the factors which best reproduce the heterogeneity in antibody responses among vaccine recipients and simultaneously estimate the correlations between antibody responses generated to the four serotypes. The application of multivariate regression models is novel in the field of immunogenicity modelling. This analysis contributes to a better understanding of the immune response conferred by CYD-TDV and assists in the biological interpretation of the phase-3 efficacy results.

## 2 Methods

### 2.1 Data

We analyse the immunogenicity data collected in the vaccine arm of five phase-2, randomised, observer-blind, controlled trials of CYD-TDV conducted in the Philippines [10], Latin America [12], Vietnam [6], Thailand [9] and Brazil [11]. The subjects enrolled in the trials were randomized with a 2:1 ratio to receive vaccine or placebo, respectively. Vaccine was delivered as three subcutaneous injections at months 0, 6 and 12. Blood samples for the assessment of the immunogenicity properties of CYD-TDV were obtained before the first dose and 28 days after each dose from all subjects in the Philippines, Latin America, Vietnam and Brazil and on a subset of 300 subjects in Thailand. Serum levels of neutralizing antibodies against each of the four CYD-TDV's dengue parental strains were determined using the 50% plaque reduction neutralizing test (PRNT50) [13]. The blood samples collected at baseline in the Philippines, Vietnam and Thailand were also tested with PRNT50 to assess Japanese encephalitis virus (JEV) seropositivity. The blood samples collected in Latin America were tested for Yellow fever virus (YFV) but due to recently suspected cross reactivity with dengue using the current assay we did not use the YFV seropositivity results in this analysis. The lower limit of quantitation of the DENV and JEV PRNT50 was 10 (1/dil) and samples with titre ≥ 10 were considered seropositive (DENV+, JEV+). We defined a categorical variable describing the DENV immune status at baseline. Following [14], subjects were defined seronegative (DENV−) if the titres against all four DENV serotypes were below 10. Subjects who were DENV seropositive at baseline were classed as having monotypic titre profiles if they had only one serotype titre ≥ 10 or more than one serotype ≥10 with a titre ≥ 80 to only one serotype and as having a multitypic profile if they had titres ≥ 10 to more than one serotype without titre ≥ 80 to only one serotype. Sensitivity analysis on the definitions of the DENV and JEV immune status is presented in the Supplementary information (SI).

### 2.2 Analysis

Initially we selected all CYD-TDV recipients with complete records of baseline (B), post-dose 1 (PD1), post-dose 2 (PD2) and post-dose 3 (PD3) titres against the four dengue serotypes and with baseline titres against YFV or JEV (see Table 1). We then examined the distribution of titres and increases in titre after each dose as a function of baseline DENV and JEV immune status.

We then developed multivariate regression models to predict PD3 titres using only information available at baseline. Table S1 details the subjects included in this analysis. The predictors considered in our analysis were: baseline titres against DENV1–4 and JEV, baseline immunological status to DENV and JEV, gender, age, trial code and trial location (see Table S2). We denote the log 10 of the PD3 titre of subject *j* (*j* = 1, ..., *n*) against DENV *i* (*i* = 1, ..., 4) as *Y*_*ji*_ and the observed *k*th *covariate* for subject *j* (*k* = 1, ..., *p*) as *W*_*jk*_. We then model the PD3 titres using multivariate linear regression models, defined by

(1) $$Y_{ji}=\sum_{k=0}^{p}W_{jk}\beta_{ki}+e_{ji}$$

where *W*_*j*0_ = 1 corresponds to an intercept, *β*_*ki*_ are the coefficients of the covariates and *e*_*ji*_ is the residual error of subject *j* against DENV serotype *i*. We assume that the errors *e*_*ji*_ are jointly normal with mean zero and that $\text{Cov}\left(e_{ji},e_{hl}\right)=0$ for different individuals *j* and *h* (i.e. individuals are independent) and $\text{Cov}\left(e_{ji},e_{jl}\right)=\Sigma_{il}$ for serotypes *i* and *l* (i.e. responses to the four DENV serotypes are correlated and the variance-covariance matrix *Σ* is the same for all subjects). We estimate the coefficients *β*_*ki*_ and the variance-covariance terms *Σ*_*il*_ by maximum likelihood and adopt the Akaike Information Criterion (AIC) for model selection and comparison. The AIC is defined as $\text{AIC}=2n_{p}-2\;\text{ln}\left(L\right)$, where *n*_*p*_ denotes the number of parameters and *L* denotes the maximized likelihood. Models with an AIC difference from the best model ΔAIC_*i*_ = AIC_*i*_ − AIC_min_ ≥ 4 are considered to have significantly less support than the best model [15].

We initially define 11 incrementally more sophisticated models using the baseline DENV antibody titres as predictors of the PD3 DENV titres. Model 1 predicts the PD3 titres using the homologous baseline titres. Models 2 and 3 extend model 1 by including the maximum or average of the heterologous baseline titres, respectively. Models 4 and 5 use all four serotype antibody titres (homologous and heterologous) as covariates; in model 4 only the coefficient of the homologous titre varies with the serotype response being predicted whilst in model 5 we additionally allow the coefficients of the heterologous baseline titres to vary with the response's serotype. Models 6–11 extend models 1–5 by including interactions between different serotype titres. We then progressively build on the two best fitting of these 11 model variants and increase complexity by successively adding each one of the remaining covariates (baseline immunological status to DENV and JEV; gender; age; trial code; trial location; baseline titre against JEV). All model variants are described in the SI (Section 1.4). Moreover, we tested the predictive power of the best models using cross-validation (Section 2.3 of the SI). The analysis was performed using MATLAB (release R2013b) and R (version 3.0.2).

## 3 Results

A total number of 867 subjects in the vaccine group had complete records of baseline, PD1, PD2 and PD3 titres against the four dengue serotypes and baseline titres against JEV. Details on the baseline demographic and immunological characteristics of the subjects included in the analysis are given in Table 1.

**Fig. 1:**
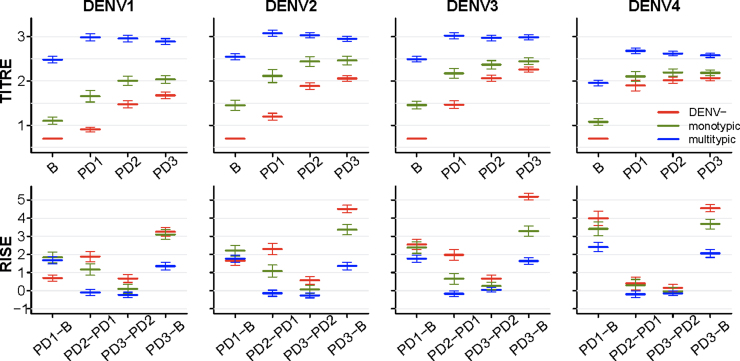
Mean and 95% CI of observed baseline (B), post-dose 1 (PD1), post-dose 2 (PD2) and post-dose 3 (PD3) titres (row 1) and rises in titres (row 2) for each DENV serotype (columns) by baseline immunological status against DENV (colour code). Undetectable titres (below the detection threshold of 10) are assigned a titre value of 5. Titres are shown on a log 10 scale. Increases in titres are shown on a log 2 scale, according to the definition.

Fig. 1 shows the mean and 95% confidence interval (CI) of the DENV titre measurements and the rises in titres observed after each vaccination, stratified by the baseline DENV immunological status of the subjects (i.e. DENV seronegative, monotypic or multitypic profile). Subjects with multitypic baseline DENV status developed significantly higher DENV titres (at PD1, PD2 and PD3 measurements) than subjects who had a seronegative or monotypic baseline DENV immune status. Subjects with monotypic profile at baseline showed PD3 titres in between DENV seronegative and multitypic subjects. Fig. 1 also shows that the observed rises in titres varied by serotype and similar trends were observed having stratified the baseline monotypic subjects into subclasses, according to the (presumed) infecting serotype (Figs. S2 and S3).

Figs. S4 and S5, respectively, show DENV titres and rises in titres after each dose, stratified by baseline DENV immunological status and continent of enrolment (i.e. Southeast Asia or Latin America), trial and JEV status. We observe (Fig. S4) that Latin American DENV seropositive subjects had significantly higher DENV1–4 titres (both pre-vaccination and at all measurement time points post-vaccination) than Southeast Asian DENV seropositive subjects. Moreover, within the group of subjects who were DENV seropositive at baseline, subjects who were also seropositive to JEV showed higher DENV1–4 titres than JEV seronegative subjects. Figs. S4 and S5 also illustrate the substantial variability in DENV titres and increases in titres observed between trials.

**Fig. 2:**
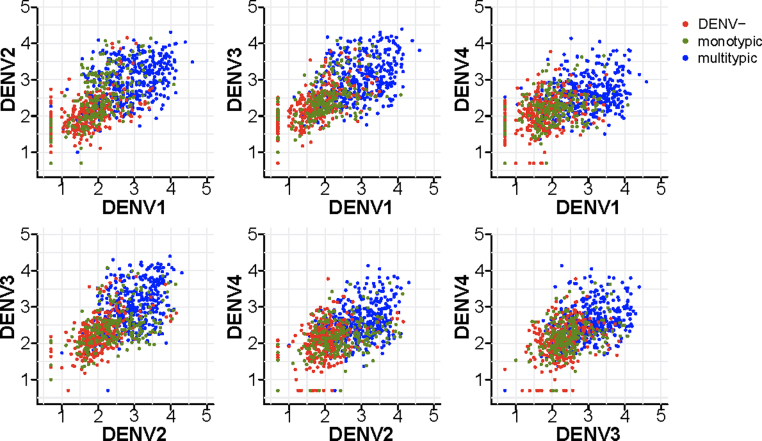
Scatterplots of post-dose 3 (PD3) titres against heterologous serotypes. Each point represents an observation and the colour of the point denotes the baseline immunological status against DENV. “DENV−” denotes DENV seronegative (i.e. baseline PRNT50 titres < 10 for all DENV serotypes), “monotypic” denotes subjects with PRNT50 titres ≥ 10 for one DENV serotype only or more than one DENV serotype with a titre ≥ 80 to only one DENV serotype, “multitypic” denotes subjects with PRNT50 titres ≥ 10 for at least two DENV serotypes without titre ≥ 80 to only one DENV serotype. Undetectable titres (below the detection threshold of 10) are assigned a titre value of 5. Titres are shown on a log 10 scale.

Fig, 2 shows pairwise scatterplots of the observed PD3 DENV titres by serotype, stratified by the baseline immunological status against DENV. The PD3 titres of the subjects with multitypic immunological profiles at baseline tend to cluster in the top right region of the plane, where the titres against both serotypes are high, those of seronegative subjects at baseline tend to cluster in the lower region of the plane, where the titres against both serotypes are low and the PD3 titres of monotypic profiles at baseline scatter in-between the two aforementioned clusters. The correlation observed in the PD3 titres against DENV1–4 (Fig. 2) together with the joint normality of the data (Fig. S1) supports the choice of a multivariate normal regression model [16].

From the analysis of regression models 1–11 (Table S3) we find that models including all (homologous and heterologous) baseline DENV titres (models 4 and 5) fit the data significantly better than models which use the homologous baseline titre as the only predictor (model 1) or which include a summary statistic of the heterologous titres (models 2 and 3). The addition of interaction terms between the homologous titre and either all heterologous titres or their average or their maximum further improves the fit and we find that the two best fitting models by AIC (models 9 and 11) are obtained by adding an interaction between the homologous titre and the maximum of the heterologous titres to model variants 4 and 5 (Table 2). Tables S4 and S7 show the results of the two best fitting models, having successively added one, two and three additional covariates. We find that adding a trial-specific intercept produces the biggest improvement in terms of AIC and explained variance; adding further information on the baseline DENV status produces the second largest improvement in AIC whilst adding the remaining covariates (gender, age, JEV immunological status and JEV titre) does not produce a substantial improvement in model fit (ΔAIC < 4) (Tables S4 and S7). The best models overall, denoted models 9j and 11j, predict the PD3 titres using all (homologous and heterologous) baseline titres, the interaction between the homologous and the maximum of the heterologous titres and include trial- and DENV status-specific intercepts (see Eqs. (13) and (15) in the SI). Table 2 summarises the goodness of fit obtained with the most representative model variants explored in the multivariate regression analysis.

**Fig. 3:**
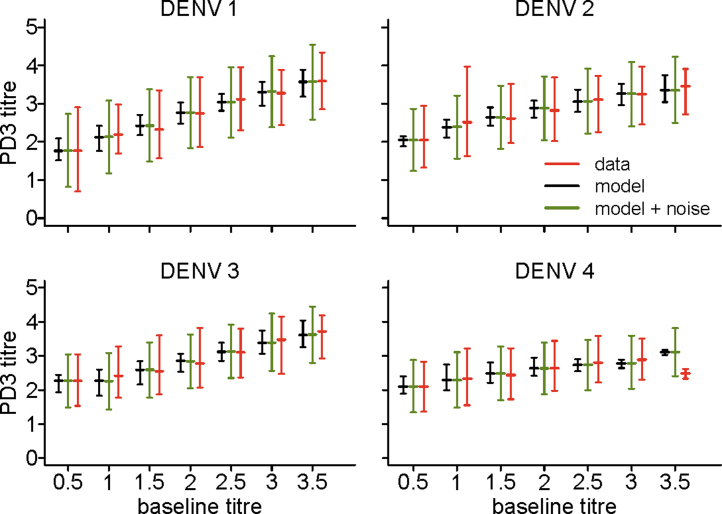
Observed (red), expected (black) and expected with simulated noise (green) mean and 5–95 percentiles of the post-dose 3 (PD3) titres versus the homologous baseline titres, obtained with the best model (model 9j). Titres are shown on a log 10 scale and baseline titres are grouped in bins of width 0.5 (the first bin representing undetectable titres at baseline).

Fig. 3 shows the fit of model 9j to the observed PD3 titres as function of the homologous baseline titre. The expected average titre predictions across all subjects are obtained using the maximum likelihood parameter estimates for model 9j (Table S5). To obtain the model's predictions with noise (in green in Fig. 3), for each subject we simulated 100 independent realizations of the error *e*_*j*_ = [*e*_*j*1_, *e*_*j*2_, *e*_*j*3_, *e*_*j*4_] from a multivariate normal distribution with mean zero and the estimated variance-covariance matrix, and then added the simulated noise to the central prediction for that subject. Fig. 3 and Fig. S6 show that model 9j not only matches the average responses but also reproduces the variability observed in the data.

The parameter estimates obtained with models 9j and 11j (Tables S5 and S8) show that the PD3 titres are significantly positively associated with the homologous baseline titre. We also find a significant negative association between the PD3 titre against DENV1–3 and the baseline titre against DENV4 (see estimates of *β*_2*i*_ and *β*_3*i*_ in Table S5 and of *β*_3*i*_ and *β*_7*i*_ in Table S8). PD3 titres are negatively associated with the interaction term between the homologous and maximum of the heterologous titres (see estimates of *β*_4*i*_ in Table S5 and of *β*_8*i*_ in Table S8), which implies that if the homologous titre or the maximum of the heterologous titres (or both) are high at baseline then the increase in titre induced by vaccination is decreased (reflecting either saturation in the level of titres that can be attained or lack of infectivity of CYD-TDV due to pre-existing antibodies). Furthermore, our analysis suggests that DENV4 titre responses to vaccination are the least correlated with other serotypes, with an estimated correlation with DENV1–3 of about 0.4; the correlation between the PD3 titres to DENV1 and DENV3 is 0.57, and to DENV1 and DENV2 and to DENV2 and DENV3 is 0.52 (Tables S6 and S9).

We assess the predictive power of the best fit models using a 2-fold cross-validation approach, and find that they each explain at least 57%, 50%, 48% and 27% of the variance observed in the PD3 titres against DENV1–DENV4, respectively (see Section 2.3 of the SI for details).

Sensitivity analysis on the definition of DENV seronegative, monotypic and multitypic (Section 3.3 of the SI) shows that using a threshold dilution of 40 instead of 10 to define seronegativity shifts the PD3 titres of the subjects with monotypic profiles at baseline upwards (Figs. S7–S12 and S17–S22). Using a threshold titre of 40 (instead of 10) to classify JEV seropositivity (Figs. S13 and S14) does not perturb the patterns observed in Figs. S4 and S5. The fact that the best model overall is obtained using the threshold dilution of 40 to define seronegativity (Table S16) suggests that there is limited variability in the response to CYD-TDV for titres below 40.

## 4 Discussion and conclusions

We have found that the PRNT50 responses elicited by the CYD-TDV vaccine vary primarily by baseline immunological status against DENV and by trial. The final PD3 DENV titres of subjects with a multitypic immunological profile at baseline are significantly higher than the titres reached by those seronegative or with monotypic baseline profiles (Fig. 1), though the increases in titres induced by vaccination are lowest in baseline multitypic subjects. Presumably B- and T-cell immune memory created by prior infection(s), results in different effector responses to vaccination, translating to different humoural response levels.

We find no evidence of differences in final PD3 titres or increases in titres by gender or age once baseline immunological status is taken into account.

There is substantial heterogeneity in the DENV titres observed across trials and we found that including a trial-specific intercept to the regression models substantially improved model fit. The estimates of the regression coefficients associated with trials T2–T5 significantly differ from the estimate associated with trial T1 (Tables S5 and S8), which may indicate a potential role of age-related factors such as maternal antibodies in the immune response elicited by CYD-TDV. However, we can exclude that the observed variation was due to lack in standardisation of the PRNT assay [17–19] since the PRNT50 assays were performed in a single laboratory for all trials under validated conditions.

We find that subjects enrolled in Latin America show considerably higher titres than subjects recruited in Southeast Asia, both at baseline and after each vaccine dose (Fig. S4, row 1). Beyond demographic and genetic factors, previous exposure to YFV could have contributed to the higher DENV titres. The positive effect of baseline immunity to YFV (following either natural infection or immunization) on the titres of neutralizing antibodies against DENV1–4 has been previously observed in monkeys and humans [20–26]. Moreover, vaccination with CYD-TDV in YFV pre-immune individuals could recall cellular responses against the YFV backbone strain, which could assist through a bystander effect on specific responses against the envelope proteins.

In agreement with [27], we find that pre-existing immunity to JEV also induces a broader and stronger response to CYD-TDV vaccination (Fig. S4, row 3). Cross-reactive JEV-DENV E-specific memory B cells could be recalled upon CYD-TDV vaccination, boosting cross-reactive responses measured in dengue PRNT.

Supporting earlier reports [24,28] we find that DENV4 shows the largest increase in antibody titre of all the serotypes after the first vaccine dose, suggesting immunodominance of this vaccine component. DENV4 antibody titres show very limited increases after the second and third doses (Fig. 1 and Fig. S5), while DENV1–3 titres increase substantially. However, it is unclear the extent to which the similar final PD3 titres seen reflects a balanced homotypic response to all four serotypes or is affected by cross-reactive heterotypic responses, notably against the immunodominant DENV4 component. Overall, PD3 DENV4 titres thus end up comparable to, if not a little lower than, titres to the other three serotypes (Fig. 1 and Fig. S4).

We find that the rises in DENV titres tend to be inversely proportional to the baseline titres (i.e. the higher the baseline titre, the lower the increase) and the second and third vaccine doses do not substantially boost the titres of subjects with a multitypic immunological profile at baseline (Fig. 1), suggesting that sterilising immunity has been induced. Similarly, our regression models show a negative coefficient for the interaction term (between homologous and heterologous titres) as a predictor of PD3 titres (Tables S5 and S8); i.e. the presence of high (either homologous or heterologous) baseline titres limits vaccine-induced increases in titres. However, in subjects who were seronegative at baseline, significant increases in titres are seen after the second vaccine dose and some additional benefit from the third (Fig. 1). This suggests that CYD-TDV does not induce complete protection in subjects who were seronegative at baseline, consistent with the efficacy profile observed in the phase-3 trials [30,31].

We found that baseline DENV4 titres are significantly negatively associated with PD3 titres against DENV1–3; i.e. the presence of high DENV4 titres limits the increase in DENV1–3 titres. Interestingly, a similar but reverse association (i.e. the presence of DENV1 and DENV3 titres limiting the increase in DENV4 titres) was observed in naturally infected subjects [17]. Serotype dominance could be a possible and consistent explanation of this negative association. Consistent with Fig. 2, we estimate that the PD3 titres against DENV1–4 are positively correlated with each other, which suggests no evidence of competition among the DENV serotypes, though DENV4 is the least correlated serotype.

We have shown that our best fitting multivariate regression models can accurately reproduce the distribution of observed post-vaccination titres using information collected at baseline (Fig. 3) and the good predictive power of these models suggests that they could be used for predicting immunological outcomes in future studies at a population level.

The statistical approach adopted in this study is novel in the field of immunogenicity modelling and can be applied to a variety of infections with interacting strains. Early results from two large-scale phase-3 clinical studies are encouraging [29–31] and the detailed analysis of the phase-3 data will provide further insight into the immunogenicity and efficacy of CYD-TDV, in particular in subjects with different levels of pre-existing immunity against DENV, YFV and JEV.

## Funding

ID, RA, CAD and NMF acknowledge research funding from the European Union EMPERIE and PREDEMICS projects, the National Institute of General Medical Sciences Models of Infectious Disease Agent Study initiative and the Bill and Melinda Gates Foundation (grants P20064 and P46908). They also thank the UK Medical Research Council for Centre funding and the UK National Institute for Health Research for Health Protection Research Unit funding.

## Conflict of interest statement

ID, RA, CAD and NMF declare no conflict of interest. NJ, LC, MS, BG are employed by Sanofi Pasteur, the manufacturer of CYD-TDV.

## Figures and Tables

**Table 1:** Baseline demographic characteristics of the subjects included in the descriptive analysis.

Study	Location	Age (years)	*N*	Females	JEV+	DENV−	DENV+ monotypic	DENV+ multitypic
T1	Philippines	1.1 (1.0, 1.2)	116	53 (46%)	6 (5%)	61 (53%)	44 (38%)	11 (9%)
T2	Latin America	12.6 (9.4, 16.2)	361	187 (52%)	–	91 (25%)	55 (15%)	215 (60%)
T3	Vietnam	10.8 (3.1, 28.3)	113	59 (52%)	42 (37%)	34 (30%)	37 (33%)	42 (37%)
T4	Thailand	8.3 (4.9, 11.0)	188	108 (57%)	151 (80%)	55 (29%)	51 (27%)	82 (44%)
T5	Brazil	12.7 (9.3, 16.3)	89	52 (58%)	–	26 (29%)	14 (16%)	49 (55%)

*Note*: Study denotes the trail identifier; Location denotes the location where the trial was conducted, i.e. the country if the trial was single-site or the region if the trial was multicentre; Age denotes the observed mean and 5–95 percentiles of the age of the subjects included in the analysis in years; *N* denotes the number of subjects; JEV+ denotes subjects with titre against JEV  ≥  10; DENV− denotes subjects with titres < 10 for all four DENV serotypes; DENV+ denotes subjects with titres  ≥  10 for at least one DENV serotype; monotypic denotes subjects with titres  ≥  10 for one DENV serotype only or more than one DENV serotype with a titre ≥ 80 to only one DENV serotype; multitypic denotes subjects with titres  ≥  10 for at least two DENV serotypes without titre ≥ 80 to only one DENV serotype. The percentages within parentheses are computed on the number of subjects in each study (*N*). All titres have been quantified using PRNT50.

**Table 2:** Summary measures of goodness of fit of selected model variants.

Model	Covariates	AIC	Log-like	np	$R_{1}^{2}$	$R_{2}^{2}$	$R_{3}^{2}$	$R_{4}^{2}$
1	*X*_*ji*_	4440.73	−2198.36	22	0.53	0.48	0.43	0.21
4	*X*_*j*1_, ..., *X*_*j*4_	4374.07	−2157.03	30	0.55	0.50	0.45	0.24
5	*X*_*j*1_, ..., *X*_*j*4_	4371.75	−2139.88	46	0.55	0.51	0.46	0.24
9	$X_{j1},...,X_{j4},X_{ji}\times \underset{k\neq i}{\max}X_{jk}$	4350.62	−2137.31	38	0.56	0.51	0.46	0.25
11	$X_{j1},...,X_{j4},X_{ji}\times \underset{k\neq i}{\max}X_{jk}$	4351.85	−2125.92	50	0.56	0.51	0.46	0.24
9g	$X_{j1},...,X_{j4},X_{ji}\times \underset{k\neq i}{\max}X_{jk},Tn_{j}$	4184.91	−2038.45	54	0.58	0.52	0.50	0.31
11g	$X_{j1},...,X_{j4},X_{ji}\times \underset{k\neq i}{\max}X_{jk},Tn_{j}$	4181.09	−2024.54	66	0.59	0.52	0.50	0.31
9j	$X_{j1},...,X_{j4},X_{ji}\times \underset{k\neq i}{\max}X_{jk},Tn_{j},P_{j}$	4180.14	−2023.07	58	0.59	0.52	0.50	0.31
11j	$X_{j1},...,X_{j4},X_{ji}\times \underset{k\neq i}{\max}X_{jk},Tn_{j},P_{j}$	4179.74	−2019.87	70	0.59	0.52	0.50	0.31

*Note*: Akaike's Information Criterion (AIC), log-likelihood (Log-like), number of parameters (np), fraction of explained variance for serotype *i* ($R_{i}^{2}$). *X*_*ji*_ represents the log 10 baseline titre of subject *j* to DENV serotype *i*; *Tn*_*j*_ denotes the trial identifier, i.e. *T*1_*j*_, *T*2_*j*_, *T*3_*j*_, *T*4_*j*_, *T*5_*j*_; *P*_*j*_ denotes whether subject *j* is seropositive to a single DENV serotype.
