# Supplementary material for: Modelling the immunological response to a tetravalent dengue vaccine from multiple phase-2 trials in Latin America and South East Asia
Source: Vaccine. 2015 Jul 17;33(31):3746–51. doi: 10.1016/j.vaccine.2015.05.059 (PMC4504002; doi:10.1016/j.vaccine.2015.05.059)
Supplement: Supplementary file 1 [file mmc1.pdf]

Supplementary Information - Modelling the immunological response to a tetravalent dengue vaccine from multiple phase-2 trials in Latin America and South East Asia

Ilaria Dorigatti*[1], Ricardo Aguas[1], Christl A. Donnelly[1], Bruno Guy[2], Laurent Coudeville[2], Nicholas Jackson[2], Melanie Saville[2] and Neil M. Ferguson[1]

1 MRC Centre for Outbreak Analysis and Modelling, Department of Infectious Disease Epidemiology, School of Public Health, Imperial College London, Norfolk Place, London W2 1PG, United Kingdom.

2 Sanofi Pasteur, 2 avenue Pont Pasteur 69367 Lyon Cedex 07, France.

* Author for correspondence: i.dorigatti@imperial.ac.uk

phone: +44 (0)20 7594 3229

Contents

1 Materials and Methods **3**

1.1 Data . . . 3

1.2 Notation . . . 3

1.3 Assessing the joint normality of the data . . . 4

1.4 Model variants analysed . . . 5

1.4.1 Modelling the PD3 DENV titres using the baseline DENV titres as covariates . . . 5

1.4.2 Building on model 9 (the best model) . . . 7

1.4.3 Building on model 11 . . . 8

2 Results **9**

2.1 Descriptive analysis . . . 9

2.2 Model comparison and parameter estimates . . . 13

2.3 Assessing the predictive power of the best fit models . . . 20

3 Sensitivity Analysis **21**

3.1 Exploring further variants of model 9 . . . 21

3.1.1 With covariates on the slope . . . 22

3.2 Exploring further variants of model 11 . . . 23

3.2.1 With covariates on the slope . . . 23

3.3 Alternative definitions of the baseline immunological status against DENV and JEV . . . 25

3.3.1 Definition 2 with threshold at 10 . . . 25

3.3.2 Definition 2 with threshold at 40 . . . 28

3.3.3 Definition 3 with threshold at 10 . . . 38

3.3.4 Definition 3 with threshold at 40 . . . 40

3.3.5 Comparison between definitions . . . 44

1 Materials and Methods

1.1 Data

In the descriptive analysis we selected all subjects in the vaccine group with complete records of baseline, post-dose 1 (PD1), post-dose 2 (PD2) and post-dose 3 (PD3) titres against the four dengue serotypes and with baseline titres against JEV (see Table 1 in the main text). In the multivariate regression analysis we included all subjects in the vaccine group with complete records of baseline and PD3 titres against all four dengue serotypes and baseline JEV titre. Table S1 describes the baseline demographic and immunological characteristics of the subjects included in the multivariate regression analysis.

Study	Location	Age (years)	N	Females	JEV+	DENV-	DENV+ monotypic	DENV+ multitypic
T1	Philippines	1.2 (1.0, 1.3)	174	74(43%)	12(7%)	98(56%)	58(33%)	18(11%)
T2	Latin America	12.6 (9.4, 16.2)	363	187(52%)	-	92(25%)	55(15%)	216(60%)
T3	Vietnam	10.8 (3.1, 28.3)	113	59(52%)	42(37%)	34(30%)	37(33%)	42(37%)
T4	Thailand	8.3 (4.9, 11.0)	188	108(57%)	151(80%)	55(29%)	51(27%)	82(44%)
T5	Brazil	12.7 (9.4, 16.3)	89	52(58%)	-	26(29%)	14(16%)	49(55%)

Table S1: Baseline demographic characteristics of the subjects included in the multivariate regression analysis. Study denotes the trial identifier; Location denotes the location where the trial was conducted, i.e. the country if the trial was single-site or the region if the trial was multicentre; Age denotes the observed mean and 5-95 percentiles of the observed age of the subjects included in the analysis in years; N denotes the number of subjects; JEV+ denotes subjects with titre against JEV ≥ 10; DENV- denotes subjects with titres < 10 for all four DENV serotypes; DENV+ denotes subjects with titre ≥ 10 for at least one DENV serotype; monotypic denotes subjects with titre ≥ 10 for one DENV serotype or titre ≥ 10 to more than one serotype with titre ≥ 80 to one DENV serotype only; multitypic denotes subjects with titre ≥ 10 for more than one DENV serotype without titre ≥ 80 to only one serotype. The percentages within parentheses are computed on the number of subjects in each study N. All titres have been quantified using PRNT50.

1.2 Notation

We denote Y_{ji} the log10 PD3 titre of subject j against DENV i and W_{jk} the set of predictors for subject j, $k = 1, \ldots, p$. The covariates used in the regression models contain immunological (baseline titres against DENV and JEV and immunological status against DENV and JEV), demographic (gender, age) and geographical (trial code, trial location) information for each subject in the study. The covariates and the notation used are described in Table S2.

Covariate	Type	Description
X_{ji}	continuous	baseline log10 titre against DENVi for subject j
U_{ji}	dummy	1 if X_{ji} undetected, 0 otherwise
Z_{ji}	dummy	1 if $U_{jk} = 1$ for all $k \neq i$
V_{ji}	dummy	1 if $U_{jk} = 0$ for all $k \neq i$
$T1_j$	dummy	1 if subject j is part of trial T1, 0 otherwise
$T2_j$	dummy	1 if subject j is part of trial T2, 0 otherwise
$T3_j$	dummy	1 if subject j is part of trial T3, 0 otherwise
$T4_j$	dummy	1 if subject j is part of trial T4, 0 otherwise
$T5_j$	dummy	1 if subject j is part of trial T5, 0 otherwise
L_j	dummy	1 if subject j is part of a Latin American trial (T2 or T5), 0 otherwise
F_j	dummy	1 if subject j is female, 0 otherwise
P_j	dummy	1 if subject j is DENV seropositive to a single DENV serotype, 0 otherwise
JE_{N_j}	dummy	1 if subject j tests seronegative to JEV at baseline, 0 otherwise
JE_{P_j}	dummy	1 if subject j test seropositive to JEV at baseline, 0 otherwise
JET_j	continuous	baseline log10 titre against JEV for subject j
A_j	continuous	age of subject j
A_{1_j}	dummy	1 if subject j is < 5 years old at baseline, 0 otherwise
A_{2_j}	dummy	1 if subject j is ≥ 5 and ≤ 10 years old at baseline, 0 otherwise
A_{3_j}	dummy	1 if subject j is > 10 years old at baseline, 0 otherwise

Table S2: Summary of variables adopted in the analysis.

1.3 Assessing the joint normality of the data

We judge the joint normality of the data through visual inspection of the chi-squared plot of the data. Denote $Y_{j\cdot}$ the sample observations for subject j, $\bar{Y}_i$ the sample mean of the PD3 titre against DENV i and $\bar{Y}$ the vector of samples means against DENV1-4. Let S denote the sample variance-covariance matrix of the observed PD3 titres. The squared generalized distances are defined

$$d_j^2 = (Y_{j\cdot} - \bar{Y})^t S^{-1} (Y_{j\cdot} - \bar{Y}), \quad j = 1, \ldots, N$$

where the superscript t denotes the transpose operation and the superscript -1 denotes the inverse operation. The chi-squared plot in Figure S1 is obtained by plotting the pairs $\left(d_{(j)}^2, \chi_p^2(\frac{j-1/2}{N})\right)$ where $d_{(j)}^2$ is the jth ordered squared distance and $\chi_p^2(\frac{j-1/2}{N})$ is the $100\frac{j-1/2}{N}$ percentile of the chi-square distribution with 4 degrees of freedom. Visual inspection of the graph in Figure S1 shows that we can assume that the distribution of the PD3 titres is multivariate normal.

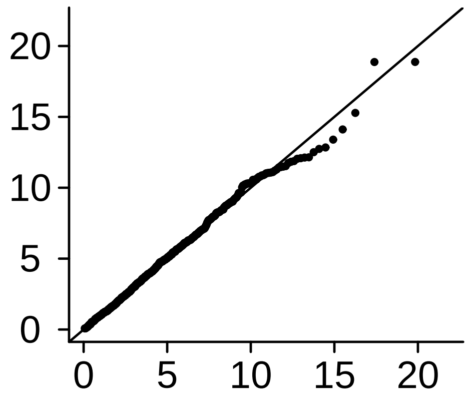

Figure S1: Chi-squared plot of the ordered distances between the PD3 titres.

1.4 Model variants analysed

In the main text (eq. (1)) we presented the general model formulation. In this section we describe all model variants explored in our analysis. The models do not depend on the value assigned to the undetected titres, since we only included the titres (or summary statistic of the titres) when these are quantified (i.e. ≥ 10) and estimate the relative contribution of the undetected titres (or summary statistics involving undetected titres) when the titres involved are below the detection limit.

1.4.1 Modelling the PD3 DENV titres using the baseline DENV titres as covariates

The models presented in this section (Models 1-11) use the baseline DENV titres as predictors of the PD3 DENV titres. Model 1 models the PD3 titre of DENVi Y_{ji} using the homologous baseline titre X_{ji} as covariate, if the homologous baseline titre is detected (i.e. if $U_{ji} = 0$):

$$Y_{ji} = \beta_{0i} + X_{ji}(1 - U_{ji})\beta_{1i} + U_{ji}\beta_{2i} + e_{ji} \tag{1}$$

The covariates used in Model 2 are the homologous baseline titre (as done in Model 1) and the maximum of the heterologous baseline titre, if detected (i.e. if $Z_{ji} = 0$):

$$Y_{ji} = \beta_{0i} + X_{ji}(1 - U_{ji})\beta_{1i} + U_{ji}\beta_{2i} + \max_{k \neq i}(X_{jk})(1 - Z_{ji})\beta_{3i} + Z_{ji}\beta_{4i} + e_{ji} \tag{2}$$

The covariates used in Model 3 are the homologous baseline titre (as done in Model 1) and the average of the heterologous titres, if all heterologous titres are detected (i.e. if $V_{ji} = 1$):

$$Y_{ji} = \beta_{0i} + X_{ji}(1 - U_{ji})\beta_{1i} + U_{ji}\beta_{2i} + \text{mean}_{k \neq i}(X_{jk})V_{ji}\beta_{3i} + (1 - V_{ji})\beta_{4i} + e_{ji} \tag{3}$$

Model 4 includes all four baseline titres as covariates. In Model 4 the coefficient of the homologous baseline titre is serotype-specific (β_{1i}) whilst the coefficients of the heterologous baseline titres do not depend on the response's serotype (β_{3k}):

$$Y_{ji} = \beta_{0i} + X_{ji}(1 - U_{ji})\beta_{1i} + U_{ji}\beta_{2i} + \sum_{k=1}^{4} X_{jk}(1 - U_{jk})\beta_{3k} + \sum_{k=1}^{4} U_{jk}\beta_{4k} + e_{ji} \tag{4}$$

Model 5 includes all four baseline titres as covariates and we estimate a response's serotype-specific coefficient for each baseline titre (β_{ki}):

$$Y_{ji} = \beta_{0i} + \sum_{k=1}^{4} X_{jk}(1 - U_{jk})\beta_{ki} + \sum_{k=1}^{4} U_{jk}\beta_{(k+4)i} \tag{5}$$

Models 6-11 extend Models 2-5 by including titres interactions. Model 6 extends Model 2 and includes the interaction between the homologous baseline titre and the maximum of the heterologous baseline titres, if the interacting terms are detected (i.e. if $U_{ji} = 0$ and $Z_{ji} = 0$):

$$\begin{aligned} Y_{ji} \; &= \; \beta_{0i} + X_{ji}(1 - U_{ji})\beta_{1i} + U_{ji}\beta_{2i} + \max_{k \neq i}(X_{jk})(1 - Z_{ji})\beta_{3i} + Z_{ji}\beta_{4i} + \\ &+ X_{ji} \max_{k \neq i}(X_{jk})(1 - U_{ji})(1 - Z_{ji})\beta_{5i} + U_{ji}Z_{ji}\beta_{6i} + e_{ji} \end{aligned} \tag{6}$$

Model 7 extends Model 3 and includes the interaction between the homologous baseline titre and the average of the heterologous baseline titres, if all titres are detected (i.e. if $U_{ji} = 0$ and $V_{ji} = 1$):

$$\begin{aligned} Y_{ji} \; &= \; \beta_{0i} + X_{ji}(1 - U_{ji})\beta_{1i} + U_{ji}\beta_{2i} + \text{mean}_{k \neq i}(X_{jk})V_{ji}\beta_{3i} + (1 - V_{ji})\beta_{4i} + \\ &+ X_{ji}\text{mean}_{k \neq i}(X_{jk})V_{ji}(1 - U_{ji})\beta_{5i} + U_{ji}(1 - V_{ji})\beta_{6i} + e_{ji} \end{aligned} \tag{7}$$

Model 8 extends Model 4 and includes all interactions between the baseline homologous and heterologous titres, if the interacting titres are detected (i.e. if $U_{ji} = 0$ and $U_{jk} = 0$ for $k \neq i$):

$$\begin{aligned} Y_{ji} \; &= \; \beta_{0i} + X_{ji}(1 - U_{ji})\beta_{1i} + U_{ji}\beta_{2i} + \sum_{k=1}^{4} X_{jk}(1 - U_{jk})\beta_{3k} + \sum_{k=1}^{4} U_{jk}\beta_{4k} + \\ &+ \sum_{k \neq i} X_{ji}X_{jk}(1 - U_{ji})(1 - U_{jk})\beta_{(4+k)i} + \sum_{k \neq i} U_{ji}U_{jk}\beta_{(8+k)i} + e_{ji} \end{aligned} \tag{8}$$

Model 9 extends Model 4 and includes the interactions between the baseline homologous titre and the maximum of the heterologous titres, if detected (i.e. if $U_{ji} = 0$ and $Z_{ji} = 0$):

$$\begin{aligned} Y_{ji} &= \beta_{0i} + X_{ji}(1-U_{ji})\beta_{1i} + U_{ji}\beta_{2i} + \sum_{k=1}^{4} X_{jk}(1-U_{jk})\beta_{3k} + \sum_{k=1}^{4} U_{jk}\beta_{4k} + \\ &\quad + X_{ji}\max_{k\neq i}(X_{jk})(1-U_{ji})(1-Z_{ji})\beta_{5i} + U_{ji}Z_{ji}\beta_{6i} + e_{ji} \end{aligned} \tag{9}$$

Model 10 extends Model 5 and includes all interactions between the baseline homologous and heterologous titres, if the interacting titres are detected (i.e. if $U_{ji} = 0$ and $U_{jk} = 0$ for $k \neq i$):

$$\begin{aligned} Y_{ji} &= \beta_{0i} + \sum_{k=1}^{4} X_{jk}(1-U_{jk})\beta_{ki} + \sum_{k=1}^{4} U_{jk}\beta_{(k+4)i} + \sum_{k\neq i} X_{ji}X_{jk}(1-U_{ji})(1-U_{jk})\beta_{(8+k)i} + \\ &\quad + \sum_{k\neq i} U_{ji}U_{jk}\beta_{(12+k)i} + e_{ji} \end{aligned} \tag{10}$$

Model 11 extends Model 5 and includes the interactions between the baseline homologous titre and the maximum of the heterologous titres, if detected (i.e. if $U_{ji} = 0$ and $Z_{ji} = 0$):

$$Y_{ji} = \beta_{0i} + \sum_{k=1}^{4} X_{jk}(1-U_{jk})\beta_{ki} + \sum_{k=1}^{4} U_{jk}\beta_{(k+4)i} + X_{ji}\max_{k\neq i}(X_{jk})(1-U_{ji})(1-Z_{ji})\beta_{9i} + e_{ji} \tag{11}$$

Note that term $U_{ji}Z_{ji}$ in equations (6) and (9) is a seronegative-specific term, i.e. $U_{ji}Z_{ji} = 1$ if and only if subject j has undetected titres to all 4 serotypes.

1.4.2 Building on model 9 (the best model)

We build on Model 9 defined in (9) and explore the effect of adding one individual covariate, other than the baseline DENV titres, to the regression model. We include each covariate additively, i.e. adding a serotype-specific intercept for each predictor. In Models 9a-9g we respectively include the gender, the age of the subject, the trial location (i.e. the continent) where the subject was enrolled, information on the baseline immunological DENV status of the subjects, the JEV titre (if detected), information on the baseline immunological status against JEV and the trial-specific identifiers.

The equation of model 9g, which includes serotype-specific intercepts for each trial, is

$$\begin{aligned} Y_{ji} = X_{ji}(1-U_{ji})\beta_{0i} + U_{ji}\beta_{1i} + \sum_{k=1}^{4} X_{jk}(1-U_{jk})\beta_{2k} + \sum_{k=1}^{4} U_{jk}\beta_{3k} + X_{ji}\max_{k\neq i}(X_{jk})(1-U_{ji})(1-Z_{ji})\beta_{4i} + \\ + U_{ji}Z_{ji}\beta_{5i} + T1_j\beta_{6i} + T2_j\beta_{7i} + T3_j\beta_{8i} + T4_j\beta_{9i} + T5_j\beta_{10i} + e_{ji} \end{aligned} \tag{12}$$

Models 9h-9l build on model 9g and include an additional individual covariate to the regression mode, specifically the gender, the age of the subject, the baseline immunological DENV status of the subjects, the JEV titre (if detected), the baseline immunological status against JEV, respectively.

The equation of model 9j, which includes serotype-specific intercepts for individuals with a single baseline seropositive DENV titre, is

$$Y_{ji} = X_{ji}(1 - U_{ji})\beta_{0i} + U_{ji}\beta_{1i} + \sum_{k=1}^{4} X_{jk}(1 - U_{jk})\beta_{2k} + \sum_{k=1}^{4} U_{jk}\beta_{3k} + X_{ji} \max_{k \neq i}(X_{jk})(1 - U_{ji})(1 - Z_{ji})\beta_{4i} + \\ + U_{ji}Z_{ji}\beta_{5i} + T1_j\beta_{6i} + T2_j\beta_{7i} + T3_j\beta_{8i} + T4_j\beta_{9i} + T5_j\beta_{10i} + P_j\beta_{11i} + e_{ji} \quad (13)$$

Models 9m-9p build on Model 9j and include the gender, the age of the subject, the JEV titre (if detected) and the baseline immunological status against JEV, respectively.

1.4.3 Building on model 11

Similarly to the analysis presented in section 1.4.2, we build on Model 11 defined in (11) and explore the effect of adding one individual covariate, other than the baseline DENV titres, to the regression model. We include the extra covariates additively, i.e. adding serotype-specific intercepts for each covariate. In Models 11a-11g we respectively include the gender, the age of the subject, the trial location where the subject was enrolled, information on the baseline immunological DENV status of the subjects, the JEV titre (if detected), information on the baseline immunological status against JEV and the trial-specific identifiers. The equation of model 11g, which includes serotype-specific intercepts for each trial, is

$$Y_{ji} = \sum_{k=1}^{4} X_{jk}(1 - U_{jk})\beta_{(k-1)i} + \sum_{k=1}^{4} U_{jk}\beta_{(k+3)i} + X_{ji} \max_{k \neq i}(X_{jk})(1 - U_{ji})(1 - Z_{ji})\beta_{8i} + \\ + T1_j\beta_{9i} + T2_j\beta_{10i} + T3_j\beta_{11i} + T4_j\beta_{12i} + T5_j\beta_{13i} + e_{ji} \quad (14)$$

Models 11h-11l build on model 11g and include an additional individual covariate to the regression mode, specifically the gender, the age of the subject, the baseline immunological DENV status of the subjects, the JEV titre (if detected), the baseline immunological status against JEV, respectively.

The equation of model 11j, which includes serotype-specific intercepts for individuals with a single baseline seropositive DENV titre, is

$$Y_{ji} = \sum_{k=1}^{4} X_{jk}(1 - U_{jk})\beta_{(k-1)i} + \sum_{k=1}^{4} U_{jk}\beta_{(k+3)i} + X_{ji} \max_{k \neq i}(X_{jk})(1 - U_{ji})(1 - Z_{ji})\beta_{8i} + \\ + T1_j\beta_{9i} + T2_j\beta_{10i} + T3_j\beta_{11i} + T4_j\beta_{12i} + T5_j\beta_{13i} + P_j\beta_{14i} + e_{ji} \quad (15)$$

Models 11m-11p build on Model 11j and include the gender, the age of the subject, the JEV titre (if detected) and the baseline immunological status against JEV, respectively.

2 Results

2.1 Descriptive analysis

Figures S2 and S3 show the mean and 95% confidence interval (CI) of the observed DENV titres in time and of the observed rises in titres between successive vaccinations respectively, having classified the subjects as seronegative, monotypic profile with detected DENV1 titre, monotypic profile with detected DENV2 titre, monotypic profile with detected DENV3 titre, monotypic profile with detected DENV4 titre or multitypic infection profile.

Figures S4 and S5 show the mean and 95% CI of the observed DENV titres in time (i.e. at baseline (B), PD1, PD2 and PD3) and of the observed rises in titres between successive vaccinations (i.e. PD1-B, PD2-PD1, PD3-PD2 and PD3-B), respectively. The results are presented for each DENV serotype (columns) stratified by baseline immunological status against DENV and continent where the trial was conducted (i.e. South East Asia (SEA) or Latin America (LA)) (row 1), by baseline immunological status against DENV and trial (row 2) and by baseline immunological status against DENV and JEV (only for South East Asian studies) (row 3). The rise r_{12} between two titres T_{t_1}, T_{t_2} taken respectively at time points t_1, t_2 is defined as the number of 2-fold serial dilutions needed to obtain T_{t_2} from T_{t_1}, i.e. $r_{12} = \log_2(T_{t_2}/T_{t_1})$.

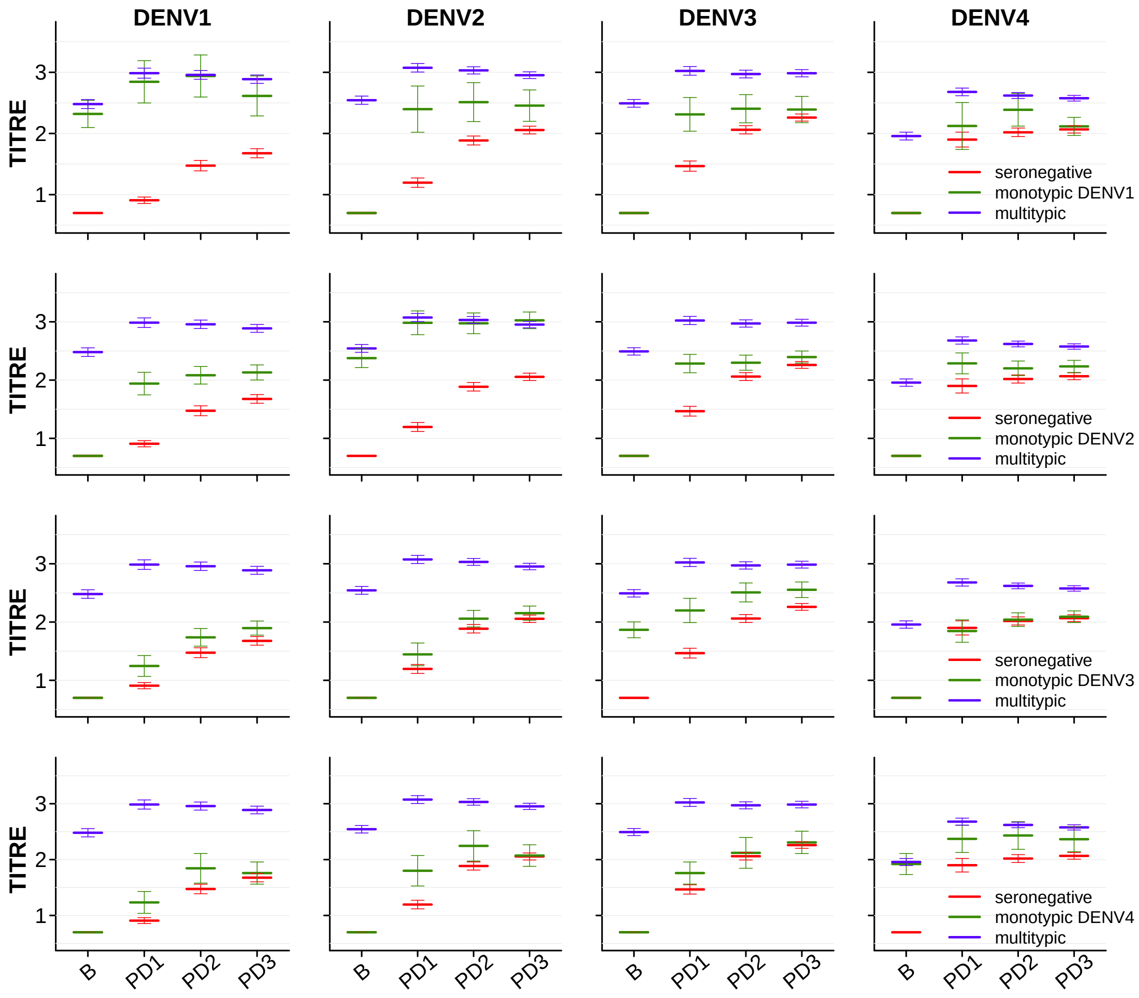

Figure S2: Mean and 95% CI of observed baseline (B), post-dose 1 (PD1), post-dose2 (PD2) and post-dose 3 (PD3) titres of seronegative, multitypic and monotypic DENV1 subjects (23/867) (row 1), monotypic DENV2 subjects (66/867) (row 2), monotypic DENV3 subjects (81/867) and monotypic DENV4 subjects (31/867). Titres are shown on a log10 scale.

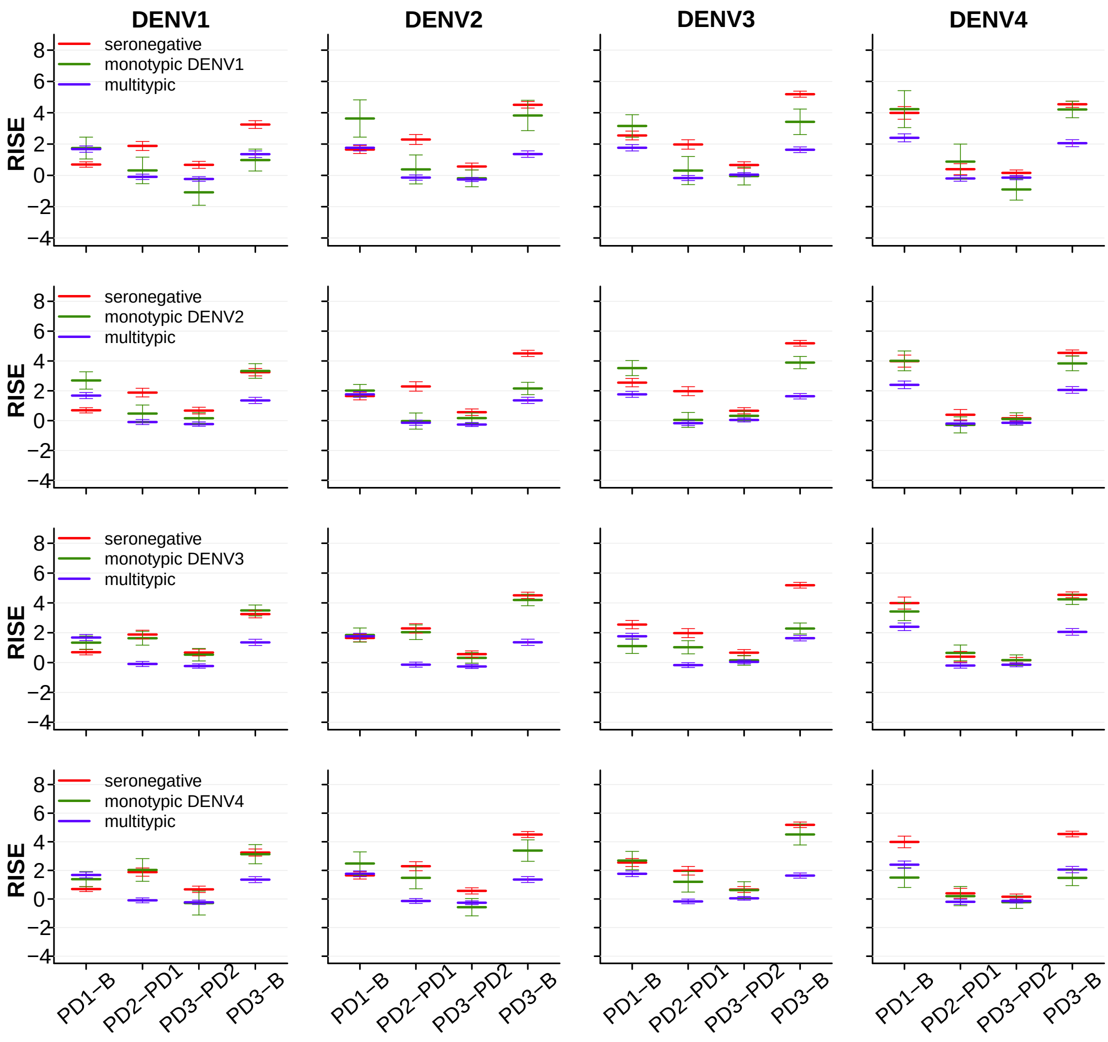

Figure S3: Mean and 95% CI of observed rises from baseline to post-dose 1 (PD1-B), from post-dose 1 to post-dose 2 (PD2-PD1), from post-dose2 to post-dose 3 (PD3-PD2) and from baseline to post-dose 3 (PD3-B) of seronegative, multitypic and monotypic DENV1 subjects (23/867) (row 1), monotypic DENV2 subjects (66/867) (row 2), monotypic DENV3 subjects (81/867) and monotypic DENV4 subjects (31/867). Increases in titres are shown on a log2 scale, according to the definition.

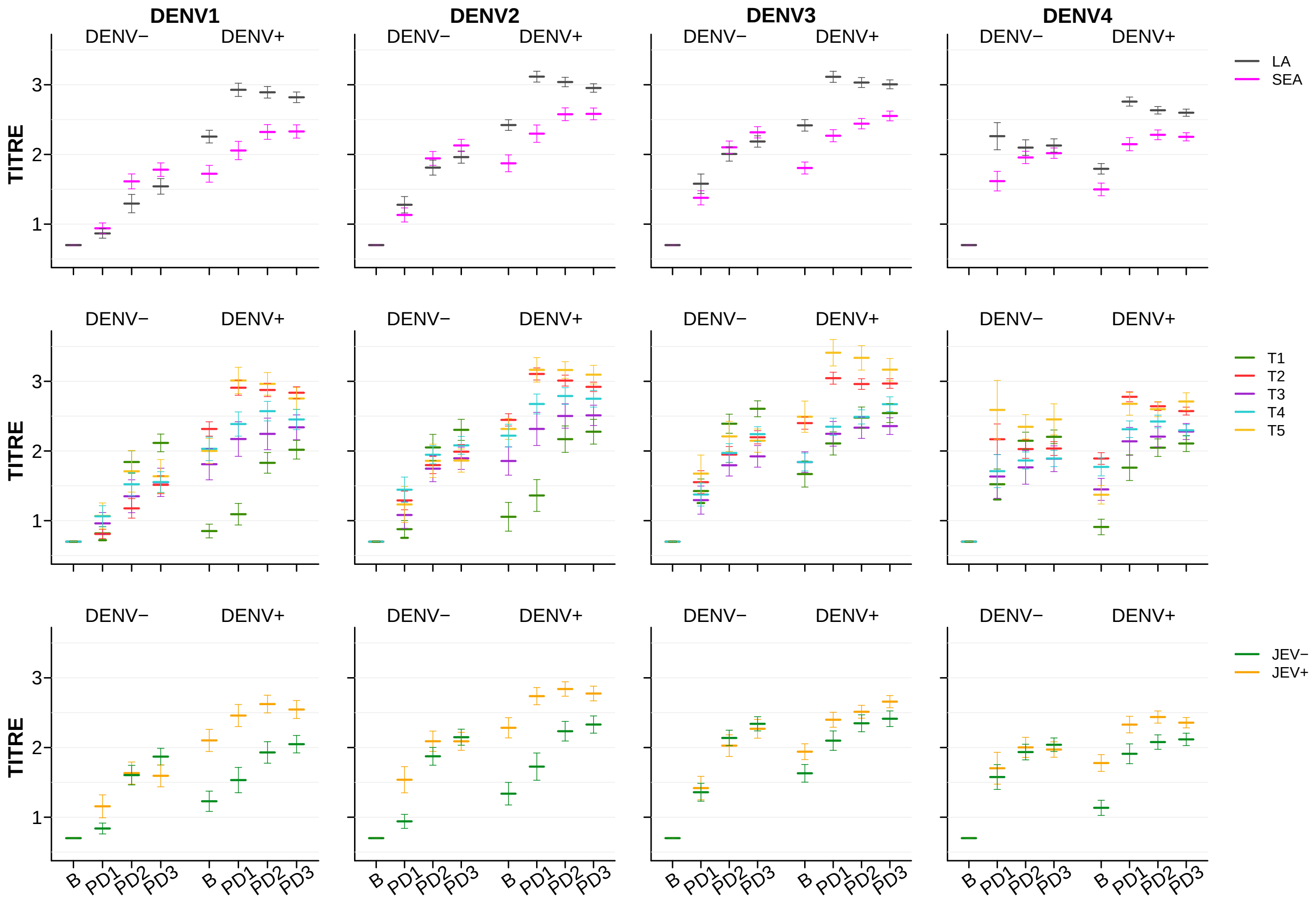

Figure S4: Mean and 95% CI of observed baseline (B), post-dose 1 (PD1), post-dose2 (PD2) and post-dose 3 (PD3) titres by baseline DENV immunological status and continent (LA=Latin America, SEA = South East Asia) (row 1), baseline DENV immunological status and study (row 2) and baseline DENV immunological status and baseline JEV immunological status (row 3). Titres are shown in a log10 scale.

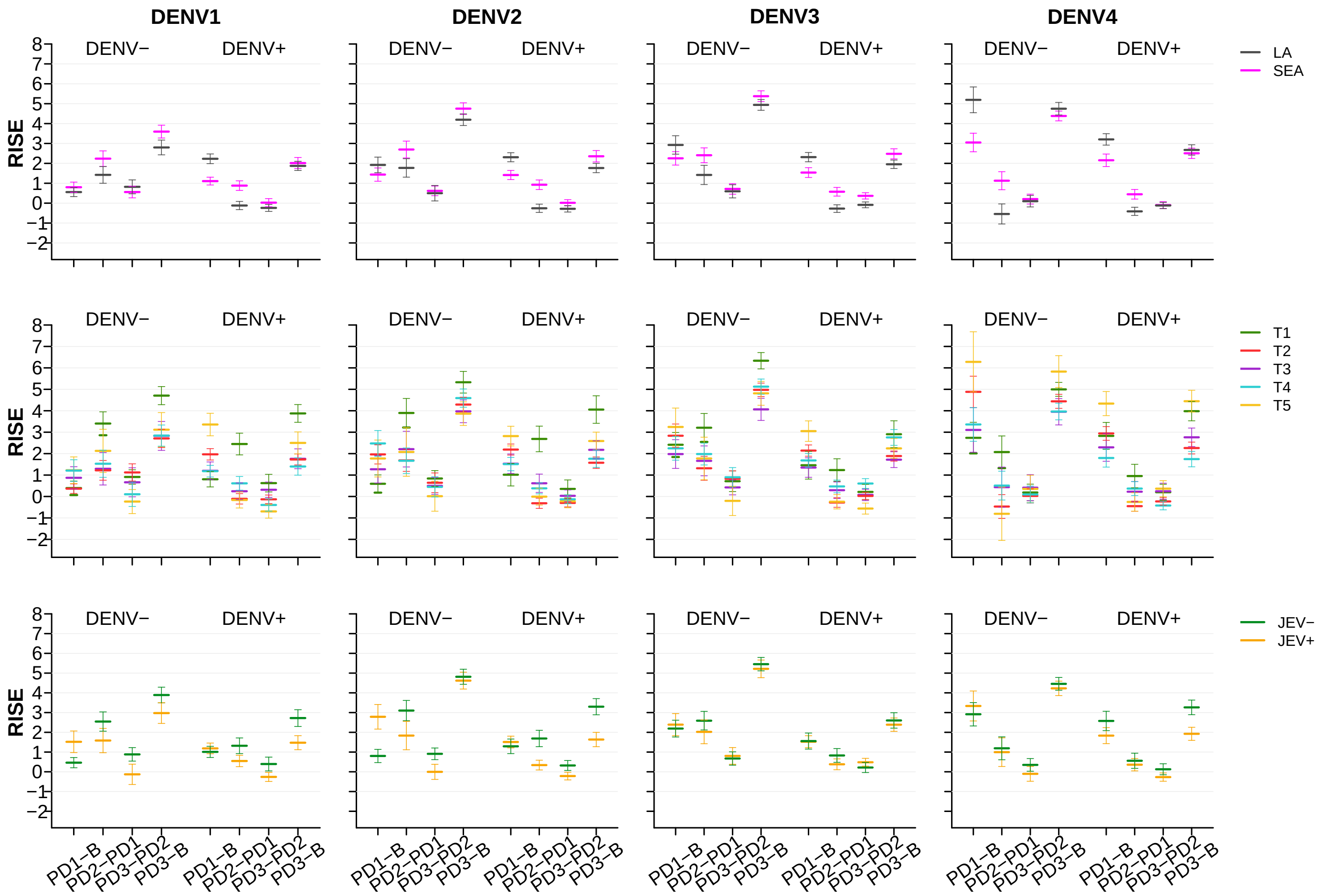

Figure S5: Mean and 95% CI of observed rises in tires between baseline and post-dose 1 (B-PD1), post-dose 1 and post-dose2 (PD1-PD2), post-dose 2 and post-dose 3 (PD2-PD3) and baseline and post-dose 3 (B-PD3) by baseline DENV immunological status and continent (LA=Latin America, SEA = South East Asia) (row 1), baseline DENV immunological status and study (row 2) and baseline DENV immunological status and baseline JEV immunological status (row 3). Increases in titres are shown on a log2 scale, according to the definition.

2.2 Model comparison and parameter estimates

The results obtained with models 1-11 are given in Table S3 and show that model 9 has the lowest AIC among models 1-11. Model 11 shows a comparable goodness of fit to model 9 ($\Delta AIC < 4$) but model 9 is more parsimonious, with 12 fewer parameters than model 11.

Table S4 shows the results, in terms of goodness of fit, obtained with model variants 9a-9p. According to the AIC, model 9j is the best model. Table S5 shows the estimates obtained with model 9j and Table S6 shows the correlation between the PD3 titres against the four DENV serotypes computed from the estimated variance-covariance terms using model 9j. The correlation ρ_{il} between the PD3 titres against the four DENV strains is computed from the maximum likelihood estimates of the variance-covariance terms $\hat{\Sigma}_{il}$ according to the definition

$$\rho_{il} = \frac{\hat{\Sigma}_{il}}{\sqrt{\hat{\Sigma}_{ii}\hat{\Sigma}_{ll}}} \tag{16}$$

Figure S6 shows the observed and expected PD3 titres versus the homologous baseline titre. The expected average PD3 titres are computed using the maximum likelihood estimates obtained with model 9j (see Table S5) and the noise is simulated as a single random realisation of the multivariate normal distribution with zero mean and the estimated variance-covariance matrix Σ using model 9j.

The results obtained with models 11a-11p are given in Table S7. Model 11m has the lowest AIC overall but the improvement in terms of AIC from model 11j is small ($\Delta AIC < 4$), hence we choose the more parsimonious model as the best fitting model. Table S8 shows the estimated parameter values obtained with model variant 11j and Table S9 shows the estimated correlation between the PD3 titres to DENV serotypes 1-4 obtained with model variant 11j.

Model	Covariates	AIC	ΔAIC	Log-like	np	R_1^2	R_2^2	R_3^2	R_4^2
1	X_{ji}	4440.73	90.10	-2198.36	22	0.53	0.48	0.43	0.21
2	X_{ji}, $\max_{k\neq i}(X_{jk})$	4415.34	64.72	-2177.67	30	0.53	0.49	0.43	0.22
3	X_{ji}, $\text{mean}_{k\neq i}(X_{jk})$	4400.14	49.52	-2170.07	30	0.54	0.49	0.44	0.23
4	$X_{j1},\ldots,X_{j4}$	4374.07	23.44	-2157.03	30	0.55	0.50	0.45	0.24
5	$X_{j1},\ldots,X_{j4}$	4371.75	21.13	-2139.88	46	0.55	0.51	0.46	0.24
6	X_{ji}, $\max_{k\neq i}(X_{jk})$, $X_{ji}\max_{k\neq i}(X_{jk})$	4379.47	28.84	-2151.73	38	0.55	0.49	0.44	0.23
7	X_{ji}, $\text{mean}_{k\neq i}(X_{jk})$, $X_{ji}\text{mean}_{k\neq i}(X_{jk})$	4391.57	40.95	-2157.78	38	0.54	0.49	0.44	0.24
8	$X_{j1},\ldots,X_{j4}$, $X_{ji}X_{jk}$ for $i\neq k$	4370.96	20.33	-2131.48	54	0.56	0.51	0.46	0.25
9	$X_{j1},\ldots,X_{j4}$, $X_{ji}\max_{k\neq i}(X_{jk})$	**4350.62**	0	-2137.31	38	0.56	0.51	0.46	0.25
10	$X_{j1},\ldots,X_{j4}$, $X_{ji}X_{jk}$ for $i\neq k$	4379.61	28.98	-2119.80	70	0.56	0.51	0.46	0.25
11	$X_{j1},\ldots,X_{j4}$, $X_{ji}\max_{k\neq i}(X_{jk})$	4351.85	1.22	-2125.92	50	0.56	0.51	0.46	0.24

Table S3: Akaike's Information Criterion score (AIC), AIC difference from best model (ΔAIC), log-likelihood (Log-like), number of parameters estimated in the regression equation (np) and fraction of serotype-specific explained variance compared to the null model ($R_1^2,\ldots,R_4^2$) obtained by fitting the observed post dose 3 titres to the specified covariates, using the regression models in equations (1)-(11). The number of estimated parameters (np) includes the coefficients of the regression models β_{ki} ($k = 0,\ldots,p$) and the variance-covariances of the serotypes Σ_{il} ($i, l = 1,\ldots,4$). The AIC of the best model is in bold.

Model	Additional covariate(s)	AIC	ΔAIC	Log-like	np	R_1^2	R_2^2	R_3^2	R_4^2
9a	F_j	4353.47	168.56	-2134.74	42	0.56	0.51	0.46	0.25
9b	A_j	4322.33	137.42	-2119.17	42	0.56	0.51	0.47	0.25
9c	L_j	4325.91	141.00	-2120.95	42	0.56	0.51	0.46	0.27
9d	P_j	4353.35	168.45	-2134.68	42	0.56	0.51	0.46	0.25
9e	JET_j	4316.92	132.01	-2112.46	46	0.56	0.51	0.45	0.27
9f	JE_{N_j}, JE_{P_j}	4319.90	134.99	-2113.95	46	0.56	0.51	0.46	0.27
9g	Ts_j $(s = 1, \ldots, 5)$	**4184.91**	0	-2038.45	54	0.58	0.52	0.50	0.31
9h	Ts_j, F_j	4185.22	5.08	-2034.61	58	0.59	0.52	0.50	0.31
9i	Ts_j, A_j	4189.86	9.72	-2036.93	58	0.58	0.52	0.50	0.31
9j	Ts_j, P_j	**4180.14**	0	-2032.07	58	0.59	0.52	0.50	0.31
9k	Ts_j, JET_j	4192.21	12.07	-2034.11	62	0.58	0.52	0.50	0.31
9l	Ts_j, JE_{N_j}, JE_{P_j}	4192.10	11.95	-2030.05	66	0.59	0.52	0.50	0.31
9j	Ts_j, P_j	4180.14	0	-2032.07	58	0.59	0.52	0.50	0.31
9m	Ts_j, P_j, F_j	4180.39	0.25	-2028.20	62	0.59	0.52	0.50	0.32
9n	Ts_j, P_j, A_j	4184.32	4.18	-2030.16	62	0.59	0.52	0.50	0.31
9o	Ts_j, P_j, JET_j	4186.82	6.67	-2031.41	62	0.59	0.52	0.50	0.31
9p	Ts_j, P_j, JE_{N_j}, JE_{P_j}	4186.85	6.71	-2023.43	70	0.59	0.52	0.50	0.32

Table S4: Akaike's Information Criterion score (AIC), AIC difference from best model (ΔAIC), log-likelihood (Log-like), number of estimated parameters (np) and fraction of serotype-specific explained variance compared to the null model ($R_1^2, \ldots, R_4^2$) obtained by fitting the observed post dose 3 titres to the baseline titres as in model 9 plus the specified covariates. Models 9a-9g, 9h-9l and 9m-9p have respectively 1,2 and 3 additional covariates compared to model 9. The AIC of the best model in each section of the table is in bold and the AIC differences are relative to the best model in each section. The number of estimated parameters (np) includes the coefficients of the regression models β_{ki} $(k = 0, \ldots, p)$ and the variance-covariances of the serotypes Σ_{il} $(i, l = 1, \ldots, 4)$.

Coefficient	Serotype i=1			Serotype i=2			Serotype i=3			Serotype i=4		
	MLE	SE	p-value	MLE	SE	p-value	MLE	SE	p-value	MLE	SE	p-value
β_{0i}	0.702	0.049	<0.001	0.607	0.045	<0.001	0.577	0.042	<0.001	0.667	0.063	<0.001
β_{1i}	0.743	0.090	<0.001	0.507	0.094	<0.001	0.799	0.085	<0.001	0.728	0.092	<0.001
β_{2i}	0.025	0.028	0.371	-0.008	0.032	0.811	0.048	0.032	0.135	-0.215	0.040	<0.001
β_{3i}	0.016	0.077	0.837	-0.213	0.088	0.016	-0.062	0.085	0.466	-0.407	0.084	<0.001
β_{4i}	-0.033	0.015	0.027	-0.058	0.013	<0.001	-0.032	0.013	0.012	-0.053	0.016	0.001
β_{5i}	-0.112	0.131	0.396	0.044	0.131	0.734	0.019	0.126	0.879	0.180	0.122	0.138
β_{6i}	1.997	0.145	<0.001	2.253	0.140	<0.001	2.292	0.132	<0.001	1.868	0.139	<0.001
β_{7i}	1.679	0.142	<0.001	2.141	0.136	<0.001	2.087	0.129	<0.001	1.843	0.138	<0.001
β_{8i}	1.557	0.144	<0.001	1.993	0.138	<0.001	1.783	0.130	<0.001	1.711	0.137	<0.001
β_{9i}	1.549	0.141	<0.001	2.116	0.135	<0.001	2.095	0.127	<0.001	1.646	0.137	<0.001
β_{10i}	1.736	0.145	<0.001	2.199	0.141	<0.001	2.104	0.134	<0.001	2.160	0.141	<0.001
β_{11i}	-0.237	0.106	0.026	-0.106	0.102	0.299	-0.174	0.096	0.070	0.038	0.091	0.676
Σ_{1i}	0.290	0.013	<0.001	0.136	0.010	<0.001	0.138	0.011	<0.001	0.090	0.009	<0.001
Σ_{2i}	0.136	0.010	<0.001	0.238	0.008	<0.001	0.111	0.009	<0.001	0.087	0.008	<0.001
Σ_{3i}	0.138	0.011	<0.001	0.111	0.009	<0.001	0.201	0.008	<0.001	0.074	0.007	<0.001
Σ_{4i}	0.090	0.009	<0.001	0.087	0.008	<0.001	0.074	0.007	<0.001	0.194	0.009	<0.001

Table S5: Maximum likelihood estimates (MLE), standard errors (SE) and two-sided p-values of the coefficients in model variant 9j. The equation of model variant 9j is $Y_{ji} = X_{ji}(1 - U_{ji})\beta_{0i} + U_{ji}\beta_{1i} + \sum_{k=1}^{4} X_{jk}(1 - U_{jk})\beta_{2k} + \sum_{k=1}^{4} U_{jk}\beta_{3k} + X_{ji} \max_{k \neq i}(X_{jk})(1 - U_{ji})(1 - Z_{ji})\beta_{4i} + U_{ji}Z_{ji}\beta_{5i} + T1_j\beta_{6i} + T2_j\beta_{7i} + T3_j\beta_{8i} + T4_j\beta_{9i} + T5_j\beta_{10i} + P_j\beta_{11i} + e_{ji}$

ρ_{il}	l=1	l=2	l=3	l=4
$i = 1$	1	0.52	0.57	0.38
$i = 2$	0.52	1	0.51	0.41
$i = 3$	0.57	0.51	1	0.37
$i = 4$	0.38	0.41	0.37	1

Table S6: Estimated correlations between the PD3 titres against DENV1-4 using model variant 9j.

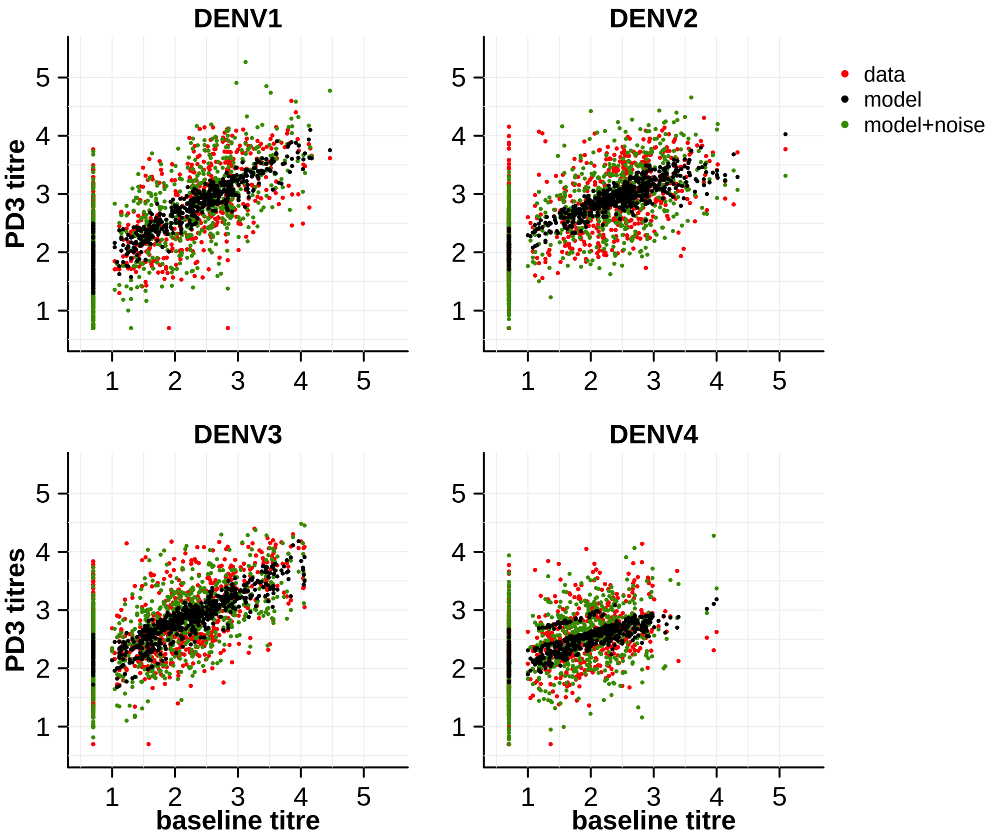

Figure S6: Observed (red), average expected (black) and expected with simulated noise (green) PD3 titres versus the observed homologous baseline titre, obtained with model 9j. The noise has been simulated by drawing a single realisation from a multivariate normal distribution with zero mean and the variance-covariance matrix Σ estimated with model 9j. Titres are shown on a log10 scale.

Model	Additional covariate(s)	AIC	ΔAIC	Log-like	np	R_1^2	R_2^2	R_3^2	R_4^2
11a	F_j	4354.23	173.15	-2123.12	54	0.56	0.51	0.46	0.25
11b	A_j	4320.94	139.85	-2106.47	54	0.56	0.51	0.47	0.25
11c	L_j	4323.18	142.09	-2107.59	54	0.56	0.51	0.46	0.27
11d	P_j	4352.70	171.61	-2122.35	54	0.56	0.51	0.46	0.25
11e	JET_j	4311.96	130.87	-2097.98	58	0.56	0.51	0.46	0.27
11f	JE_{N_j}, JE_{P_j}	4315.26	134.17	-2099.63	58	0.56	0.51	0.46	0.27
11g	Ts_j $(s = 1, \dots, 5)$	**4181.09**	0	-2024.54	66	0.59	0.52	0.50	0.31
11h	Ts_j,F_j	4180.87	1.13	-2020.44	70	0.59	0.52	0.50	0.31
11i	Ts_j,A_j	4186.22	6.48	-2023.11	70	0.59	0.52	0.50	0.31
11j	Ts_j,P_j	**4179.74**	0	-2019.87	70	0.59	0.52	0.50	0.31
11k	Ts_j,JET_j	4187.32	7.58	-2019.66	74	0.59	0.52	0.50	0.31
11l	Ts_j,JE_{N_j}, JE_{P_j}	4186.37	6.63	-2015.19	78	0.59	0.52	0.51	0.31
11j	Ts_j, P_j	4179.74	0.18	-2019.87	70	0.59	0.52	0.50	0.31
11m	Ts_j, P_j, F_j	4179.56	0	-2015.78	74	0.59	0.52	0.51	0.31
11n	Ts_j, P_j, A_j	4184.58	5.02	-2018.29	74	0.59	0.52	0.51	0.31
11o	Ts_j, P_j, JET_j	4186.60	7.04	-2019.30	74	0.59	0.52	0.50	0.31
11p	Ts_j, P_j, JE_{N_j}, JE_{P_j}	4185.51	5.94	-2010.75	82	0.59	0.52	0.51	0.31

Table S7: Akaike's Information Criterion score (AIC), AIC difference from best model (ΔAIC), log-likelihood (Log-like), number of estimated parameters (np) and fraction of serotype-specific explained variance compared to the null model ($R_1^2, \dots, R_4^2$) obtained by fitting the observed post dose 3 titres to the baseline titres as in model 11 plus the specified covariates. Models 11a-11g, 11h-11l and 11m-11p have respectively 1,2 and 3 additional covariates compared to model 11. The AIC of the best model in each section of the table is in bold and the AIC differences are relative to the best model in each section. The number of estimated parameters (np) includes the coefficients of the regression models β_{ki} $(k = 0, \dots, p)$ and the variance-covariances of the serotypes Σ_{il} $(i, l = 1, \dots, 4)$.

Coefficient	Serotype i=1			Serotype i=2			Serotype i=3			Serotype i=4		
	MLE	SE	p-value	MLE	SE	p-value	MLE	SE	p-value	MLE	SE	p-value
β_{0i}	0.824	0.070	<0.001	0.065	0.046	0.160	-0.047	0.039	0.226	0.027	0.034	0.424
β_{1i}	0.059	0.055	0.279	0.614	0.063	<0.001	-0.092	0.044	0.037	0.002	0.039	0.952
β_{2i}	0.114	0.052	0.028	0.042	0.050	0.400	0.537	0.054	<0.001	0.039	0.037	0.297
β_{3i}	-0.156	0.053	0.003	-0.246	0.049	<0.001	-0.218	0.044	<0.001	0.443	0.066	<0.001
β_{4i}	0.854	0.114	<0.001	0.064	0.110	0.559	-0.065	0.097	0.502	0.019	0.092	0.839
β_{5i}	-0.203	0.122	0.097	0.313	0.105	0.003	-0.311	0.093	<0.001	-0.109	0.093	0.242
β_{6i}	0.006	0.106	0.955	-0.012	0.094	0.895	0.651	0.086	<0.001	-0.001	0.078	0.988
β_{7i}	-0.364	0.107	<0.001	-0.460	0.098	<0.001	-0.383	0.088	<0.001	0.353	0.091	<0.001
β_{8i}	-0.082	0.024	<0.001	-0.063	0.022	0.004	0.017	0.018	0.363	-0.047	0.019	0.011
β_{9i}	1.669	0.216	<0.001	2.237	0.199	<0.001	2.548	0.162	<0.001	1.840	0.157	<0.001
β_{10i}	1.332	0.217	<0.001	2.119	0.198	<0.001	2.351	0.160	<0.001	1.818	0.156	<0.001
β_{11i}	1.233	0.213	<0.001	1.970	0.197	<0.001	2.045	0.159	<0.001	1.680	0.154	<0.001
β_{12i}	1.216	0.211	<0.001	2.091	0.193	<0.001	2.358	0.157	<0.001	1.617	0.154	<0.001
β_{13i}	1.405	0.217	<0.001	2.176	0.200	<0.001	2.368	0.164	<0.001	2.136	0.160	<0.001
β_{14i}	-0.170	0.064	0.008	-0.119	0.059	0.045	-0.151	0.056	0.007	-0.067	0.054	0.210
Σ_{1i}	0.288	0.013	<0.001	0.136	0.010	<0.001	0.138	0.011	<0.001	0.090	0.009	<0.001
Σ_{2i}	0.136	0.010	<0.002	0.237	0.008	<0.002	0.110	0.009	<0.002	0.087	0.008	<0.002
Σ_{3i}	0.138	0.011	<0.003	0.110	0.009	<0.003	0.199	0.008	<0.003	0.073	0.007	<0.003
Σ_{4i}	0.090	0.009	<0.004	0.087	0.008	<0.004	0.073	0.007	<0.004	0.194	0.009	<0.004

Table S8: Maximum likelihood estimates (MLE), standard errors (SE) and two-sided p-values of the coefficients of model variant 11j. The equation of model variant 11j is $Y_{ji} = \sum_{k=1}^{4} X_{jk}(1 - U_{jk})\beta_{(k-1)i} + \sum_{k=1}^{4} U_{jk}\beta_{(k+3)i} + X_{ji} \max_{k\neq i}(X_{jk})(1 - U_{ji})(1 - Z_{ji})\beta_{8i} + T1_j\beta_{9i} + T2_j\beta_{10i} + T3_j\beta_{11i} + T4_j\beta_{12i} + T5_j\beta_{13i} + P_j\beta_{14i} + e_{ji}$.

ρ_{il}	l=1	l=2	l=3	l=4
$i = 1$	1	0.52	0.57	0.38
$i = 2$	0.52	1	0.51	0.41
$i = 3$	0.57	0.51	1	0.37
$i = 4$	0.38	0.41	0.37	1

Table S9: Estimated correlations between the PD3 titres against DENV1-4 using model variant 11j.

2.3 Assessing the predictive power of the best fit models

We adopt a 2-fold cross-validation approach and assess the predictive power of the best models (model variants 9j and 11j) by repeating 100 times the following procedure:

1. randomly split the observed data into training T and validation V sets of indexes of equal sizes

2. fit the model to the training dataset T

3. using the MLE obtained in step 2. compute $\hat{Y}_{ji}$, the average predictions in the validation set V

4. compute a serotype-dependent R_i^2 for the training and validation sets, given by

$$R_i^2 = 1 - \frac{\sum_{j \in K}(Y_{ji} - \hat{Y}_{ji})^2}{\sum_{j \in K}(Y_{ji} - \bar{Y}_i)^2}$$

   where $K = T$ or $K = V$ and $\bar{Y}_i$ denotes the average for serotype i obtained in the training dataset T

5. swap the training and validation sets and repeat steps 2.-4.

We finally compute the average R_i^2 obtained in the 100 iterations of steps 1-5 for the training and validation sets. Table S10 summarizes the results obtained using model variants 9j and 11j.

Model	Set	R_1^2	R_2^2	R_3^2	R_4^2
9j	Training	0.60	0.53	0.51	0.32
9j	Validation	0.58	0.51	0.49	0.30
11j	Training	0.60	0.53	0.51	0.32
11j	Validation	0.57	0.50	0.48	0.27

Table S10: Average R^2s obtained in the 2-fold cross-validation analysis using model variants 9j and 11j.

3 Sensitivity Analysis

3.1 Exploring further variants of model 9

In this section we explore the effect of omitting a seronegative-specific intercept and introducing a serotype-specific intercept for profiles with a single seropositive DENV titre in model 9, given in equation (9). The equation of model 9 without a seronegative-specific intercept ($U_{ji}Z_{ji}$) is

$$Y_{ji} = \beta_{0i} + X_{ji}(1-U_{ji})\beta_{1i} + U_{ji}\beta_{2i} + \sum_{k=1}^{4} X_{jk}(1-U_{jk})\beta_{3k} + \sum_{k=1}^{4} U_{jk}\beta_{4k} + \\ + X_{ji}\max_{k\neq i}(X_{jk})(1-U_{ji})(1-Z_{ji})\beta_{5i} + e_{ji} \quad (17)$$

The equation of model 9 with a serotype-specific intercept for the profiles with a single detected titre against the homologous DENV serotype i (Z_{ji}) is

$$Y_{ji} = \beta_{0i} + X_{ji}(1-U_{ji})\beta_{1i} + U_{ji}\beta_{2i} + \sum_{k=1}^{4} X_{jk}(1-U_{jk})\beta_{3k} + \sum_{k=1}^{4} U_{jk}\beta_{4k} + \\ + X_{ji}\max_{k\neq i}(X_{jk})(1-U_{ji})(1-Z_{ji})\beta_{5i} + U_{ji}Z_{ji}\beta_{6i} + Z_{ji}\beta_{7i} + e_{ji} \quad (18)$$

The equation of model 9 having omitted $U_{ji}Z_{ji}$ and added Z_{ji} is:

$$Y_{ji} = \beta_{0i} + X_{ji}(1-U_{ji})\beta_{1i} + U_{ji}\beta_{2i} + \sum_{k=1}^{4} X_{jk}(1-U_{jk})\beta_{3k} + \sum_{k=1}^{4} U_{jk}\beta_{4k} + \\ + X_{ji}\max_{k\neq i}(X_{jk})(1-U_{ji})(1-Z_{ji})\beta_{5i} + Z_{ji}\beta_{6i} + e_{ji} \quad (19)$$

The results obtained with the variants of model 9 given in equations (17)-(19) are presented in Table S11. We find that the omission of a seronegative-specific intercept significantly worsens the fit of model 9, whilst the introduction of term Z_{ji} does not significantly improve the fit of the model to the data.

Model	Equation	AIC	ΔAIC	Log-like	np	R_1^2	R_2^2	R_3^2	R_4^2
9	see eq. (9)	4350.62	0.65	-2137.31	38	0.56	0.51	0.46	0.25
9 $-U_{ji}Z_{ji}$	see eq. (17)	4356.34	6.37	-2144.17	34	0.55	0.51	0.45	0.24
9 $+Z_{ji}$	see eq. (18)	4349.97	0	-2132.98	42	0.56	0.51	0.45	0.25
9 $-U_{ji}Z_{ji}+Z_{ji}$	see eq. (19)	4356.29	6.32	-2140.15	38	0.55	0.51	0.45	0.24

Table S11: Akaike's Information Criterion score (AIC), AIC difference from best model (ΔAIC), log-likelihood (Log-like), number of estimated parameters (np) and fraction of serotype-specific explained variance compared to the null model ($R_1^2, \ldots, R_4^2$) obtained by fitting the observed post dose 3 titres to Models given in equations (9) and (17)-(19). The number of estimated parameters (np) includes the coefficients of the regression models β_{ki} ($k = 0, \ldots, p$) and the variance-covariances of the serotypes Σ_{il} ($i, l = 1, \ldots, 4$).

3.1.1 With covariates on the slope

In this section we test the effect of including in model 9 each individual covariate multiplicatively, i.e. on the slope rather than on the intercept. This means that the covariates interact with the baseline titres, if detected. For example, when introducing the gender on the slope, we estimate separate coefficients of the baseline (detected) titres for males and females. We denote the models with covariates on the slope models $9S_1$-$9S_5$. The covariates included in models $9S_1$-$9S_5$ are respectively the gender, the trial location (i.e. the continent where the trial was conducted), the age (classified into three categorical classes $< 5, 5-10, > 10$ years), the baseline immunological status against JEV and the trial-specific identifiers. Model $9S_5$, which includes the trial identifier on the slope, estimates a separate coefficients for the baseline (detected) titres for each trial. The equation of model $9S_5$ is given by

$$
\begin{aligned}
Y_{ji} &= \beta_{0i} + X_{ji}(1-U_{ji})T1_j\beta_{1i} + X_{ji}(1-U_{ji})T2_j\beta_{2i} + X_{ji}(1-U_{ji})T3_j\beta_{3i} + \\
&+ X_{ji}(1-U_{ji})T4_j\beta_{4i} + X_{ji}(1-U_{ji})T5_j\beta_{5i} + U_{ji}\beta_{6i} + \sum_{k=1}^{4} X_{jk}(1-U_{jk})T1_j\beta_{7k} + \\
&+ \sum_{k=1}^{4} X_{jk}(1-U_{jk})T2_j\beta_{8k} + \sum_{k=1}^{4} X_{jk}(1-U_{jk})T3_j\beta_{9k} + \sum_{k=1}^{4} X_{jk}(1-U_{jk})T4_j\beta_{10k} + \\
&+ \sum_{k=1}^{4} X_{jk}(1-U_{jk})T5_j\beta_{11k} + \sum_{k=1}^{4} U_{jk}\beta_{12k} + X_{ji}\max_{k\neq i}(X_{jk})(1-U_{ji})(1-Z_{ji})\beta_{13i} + U_{ji}Z_{ji}\beta_{14i} + e_{ji}
\end{aligned}
\tag{20}
$$

Table S12 summarizes the results obtained with model variants $9S_1$-$9S_5$ and, for convenience of comparison, also of model 9. Introducing study-specific slopes improve model 9 but the improvement is much smaller than the one obtained introducing study-specific intercepts (i.e. using model 9g, see Table S4). For this reason we did not extend the analysis with models $9S_1$-$9S_5$ further.

Model	Covariates on the slope	AIC	ΔAIC	Log-like	np	R_1^2	R_2^2	R_3^2	R_4^2
9	-	4350.62	22.21	-2137.31	38	0.56	0.51	0.46	0.25
$9S_1$	F_j	4354.94	26.53	-2131.47	46	0.56	0.51	0.46	0.25
$9S_2$	L_j	4337.38	8.97	-2122.69	46	0.56	0.51	0.46	0.27
$9S_3$	A_{1_j}, A_{2_j}, A_{3_j}	4346.51	18.10	-2119.25	54	0.56	0.51	0.47	0.26
$9S_4$	JEV_{N_j}, JEV_{P_j}	4348.95	20.54	-2120.48	54	0.56	0.51	0.46	0.27
$9S_5$	Ts $(s = 1, \dots, 5)$	4328.41	0	-2094.21	70	0.56	0.52	0.47	0.28

Table S12: Akaike's Information Criterion score (AIC), AIC difference from best model (ΔAIC), log-likelihood (Log-like), number of estimated parameters (np) and fraction of serotype-specific explained variance compared to the null model ($R_1^2, \dots, R_4^2$) obtained by fitting the observed post dose 3 titres to Models 9 and $9S_1$-$9S_5$. The number of estimated parameters (np) includes the coefficients of the regression models β_{ki} $(k = 0, \dots, p)$ and the variance-covariances of the serotypes Σ_{il} $(i, l = 1, \dots, 4)$.

3.2 Exploring further variants of model 11

In this section we explore the effect of adding, to model variant 11, further information on the immunological status against DENV. Model 11 with a seronegative-specific intercept ($U_{ji}Z_{ji}$) is given by

$$Y_{ji} = \beta_{0i} + \sum_{k=1}^{4} X_{jk}(1-U_{jk})\beta_{ki} + \sum_{k=1}^{4} U_{jk}\beta_{(k+4)i} + X_{ji}\max_{k\neq i}(X_{jk})(1-U_{ji})(1-Z_{ji})\beta_{9i} + U_{ji}Z_{ji}\beta_{10i} + e_{ji} \quad (21)$$

The equation of model 11 with the extra covariate Z_{ji}, which is specific to the subjects with undetected heterologous titres ($k \neq i$), is

$$Y_{ji} = \beta_{0i} + \sum_{k=1}^{4} X_{jk}(1-U_{jk})\beta_{ki} + \sum_{k=1}^{4} U_{jk}\beta_{(k+4)i} + X_{ji}\max_{k\neq i}(X_{jk})(1-U_{ji})(1-Z_{ji})\beta_{9i} + Z_{ji}\beta_{10i} + e_{ji} \quad (22)$$

The equation of model 11 with the addition of a seronegative-specific intercept $U_{ji}Z_{ji}$ and a serotype-specific intercept for the profiles with a single detected titre against the homologous DENV serotype i (Z_{ji}) is

$$Y_{ji} = \beta_{0i} + \sum_{k=1}^{4} X_{jk}(1-U_{jk})\beta_{ki} + \sum_{k=1}^{4} U_{jk}\beta_{(k+4)i} + X_{ji}\max_{k\neq i}(X_{jk})(1-U_{ji})(1-Z_{ji})\beta_{9i} + \\ + U_{ji}Z_{ji}\beta_{10i} + Z_{ji}\beta_{11i} + e_{ji} \quad (23)$$

Table S13 summarises the results obtained with the variants of model 11 given in equations (21)-(23). The results in Table S13 show that models given in (21)-(23) do not improve the model fit.

Model	Equation	AIC	ΔAIC	Log-like	np	R_1^2	R_2^2	R_3^2	R_4^2
11	see eq. (11)	4351.85	0	-2125.92	50	0.56	0.51	0.46	0.24
11 $+U_{ji}Z_{ji}$	see eq. (21)	4351.88	0.03	-2121.94	54	0.56	0.51	0.46	0.25
11 $+Z_{ji}$	see eq. (22)	4359.58	7.73	-2125.79	54	0.56	0.51	0.46	0.24
11 $+U_{ji}Z_{ji}+Z_{ji}$	see eq. (23)	4352.16	0.31	-2118.08	58	0.56	0.51	0.46	0.25

Table S13: Akaike's Information Criterion score (AIC), AIC difference from best model (ΔAIC), log-likelihood (Log-like), number of estimated parameters (np) and fraction of serotype-specific explained variance compared to the null model ($R_1^2, \ldots, R_4^2$) obtained by fitting the observed post dose 3 titres to models given in equations (11) and (21)-(23). The number of estimated parameters (np) includes the coefficients of the regression models β_{ki} ($k = 0, \ldots, p$) and the variance-covariances of the serotypes Σ_{il} ($i, l = 1, \ldots, 4$).

3.2.1 With covariates on the slope

Similarly to the analysis presented in section 3.1.1, here we test the effect of including in model 11 each individual covariate multiplicatively, i.e. on the slope rather than on the intercept. We denote the variants of model 11 with covariates on the slope models $11S_1$-$11S_5$. The covariates included in models $11S_1$-$11S_5$ are respectively the gender, the trial location (i.e. the continent where the trial was conducted), the age

(classified into three categorical classes $< 5, 5-10, > 10$ years), the baseline immunological status against JEV and the trial-specific identifiers. Model $11S_5$, which includes the trial identifier on the slope, estimates a separate coefficients for the baseline (detected) titres for each trial. The equation of model $11S_5$ is given by

$$\begin{aligned} Y_{ji} = \beta_{0i} &+ \sum_{k=1}^{4} X_{jk}(1-U_{jk})T1_j\beta_{ki} + \sum_{k=1}^{4} X_{jk}(1-U_{jk})T2_j\beta_{(k+4)i} + \\ &+ \sum_{k=1}^{4} X_{jk}(1-U_{jk})T3_j\beta_{(k+8)i} + \sum_{k=1}^{4} X_{jk}(1-U_{jk})T4_j\beta_{(k+12)i} + \\ &+ \sum_{k=1}^{4} X_{jk}(1-U_{jk})T5_j\beta_{(k+16)i} + \sum_{k=1}^{4} U_{jk}\beta_{(k+20)i} + X_{ji}\max_{k\neq i}(X_{jk})(1-U_{ji})(1-Z_{ji})\beta_{25i} + e_{ji} \end{aligned} \quad (24)$$

Table S14 summarizes the results obtained with model variants $11S_1$-$11S_5$ and, for convenience of comparison, also of model 11. Introducing the continent-specific identifier improves model 11 but the improvement is considerably smaller than the one obtained through the introduction of trial-specific intercepts (i.e. using model 11g, see Table S7). For this reason we did not extend the analysis using model variants $11S_1$-$11S_5$ further.

Model	Covariates on the slope	AIC	ΔAIC	Log-like	np	R_1^2	R_2^2	R_3^2	R_4^2
11	-	4351.85	23.61	-2125.92	50	0.56	0.51	0.46	0.24
$11S_1$	F_J	4362.68	34.44	-2115.34	66	0.56	0.51	0.46	0.25
$11S_2$	L_j	4328.24	0	-2098.12	66	0.56	0.51	0.47	0.27
$11S_3$	$A1_j$, $A2_j$, $A3_j$	4352.04	23.80	-2094.02	82	0.56	0.51	0.47	0.26
$11S_4$	JEV_{N_j}, JEV_{P_j}	4351.68	23.44	-2093.84	82	0.56	0.51	0.47	0.27
$11S_5$	Ts $(s = 1, \dots, 5)$	4351.29	23.05	-2061.64	114	0.57	0.52	0.48	0.29

Table S14: Akaike's Information Criterion score (AIC), AIC difference from best model (ΔAIC), log-likelihood (Log-like), number of estimated parameters (np) and fraction of serotype-specific explained variance compared to the null model ($R_1^2, \dots, R_4^2$) obtained by fitting the observed post dose 3 titres to Models 11 and $11S_1$-$11S_5$. The number of estimated parameters (np) includes the coefficients of the regression models β_{ki} $(k = 0, \dots, p)$ and the variance-covariances of the serotypes Σ_{il} $(i, l = 1, \dots, 4)$.

3.3 Alternative definitions of the baseline immunological status against DENV and JEV

In this section we present the results obtained using alternative definitions of DENV seronegative, monotypic and multitypic, JEV seronegative and JEV seropositive to the ones adopted in the manuscript. The definition adopted in the manuscript, here referred as definition 1, classifies the subjects with titres < 10 to all 4 DENV serotpyes as seronegative, subjects with titre ≥ 10 to a single DENV serotype or with titre ≥ 10 to more than one DENV serotype with a titre ≥ 80 to only one serotype as monotypic and subjects with titre ≥ 10 to more than one DENV serotype without titre ≥ 80 to a single serotype as multitypic.

3.3.1 Definition 2 with threshold at 10

In this section we adopt the following definitions: an antibody pattern is classified as seronegative if it has titre < 10 to all 4 DENV serotpyes, monotypic if it has titre ≥ 10 to one DENV serotype only and multitypic if it has titre ≥ 10 to more than one DENV serotypes. We define a JEV titre < 10 to be seronegative (JEV-) and a JEV titre ≥ 10 to be seropositive (JEV+). Figure S7 shows the mean and 95% confidence interval (CI) of the observed DENV titres and raises in titres in time in DENV seronegative, monotypic and multitypic profiles, according to the definition given in this section. Figures S8 and S9 show the mean and 95% confidence interval (CI) of the observed DENV titres in time and of the observed rises in titres between successive vaccinations respectively, having classified the subjects as DENV seronegative, monotypic profile with detected DENV1 titre, monotypic profile with detected DENV2 titre, monotypic profile with detected DENV3 titre, monotypic profile with detected DENV4 titre or multitypic infection profile, according to the definition given in this section.

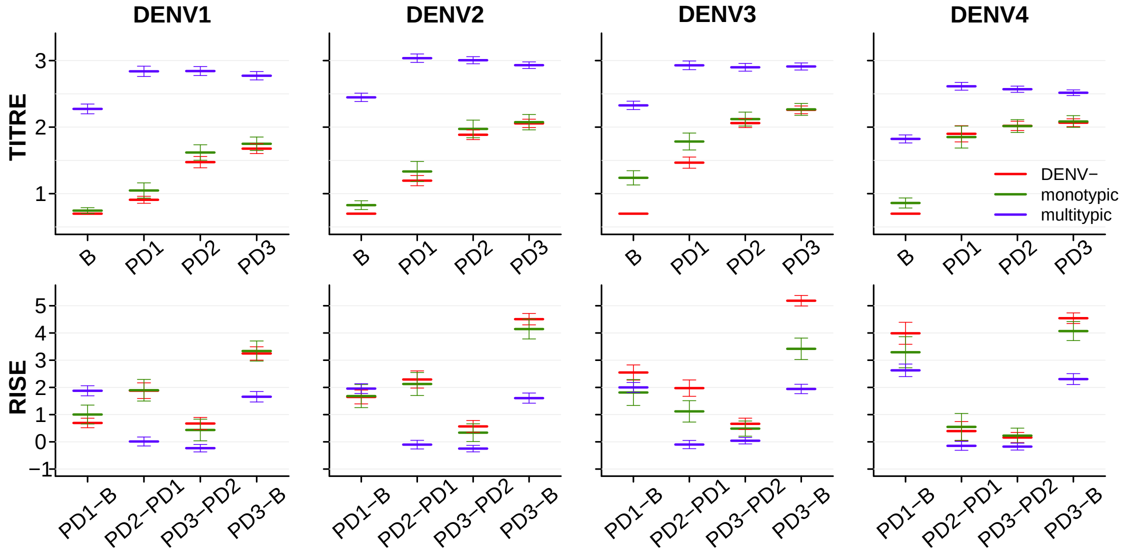

Figure S7: Mean and 95% CI of observed baseline (B), post-dose 1 (PD1), post-dose 2 (PD2) and post-dose 3 (PD3) titres (row 1) and rises in titres (row 2) for each DENV serotype (columns) by baseline immunological status against DENV (colour code). Here, DENV- denotes subjects with titre <10 for all DENV serotypes; monotypic denotes subjects with only one serotype titre $\geq$ 10; multitypic denotes subjects with titre $\geq$ 10 for at least two DENV serotypes. Undetectable titres (i.e. titres <10) are assigned a titre value of 5. Titres are shown on a log10 scale. Increases in titres are shown on a log2 scale, according to the definition.

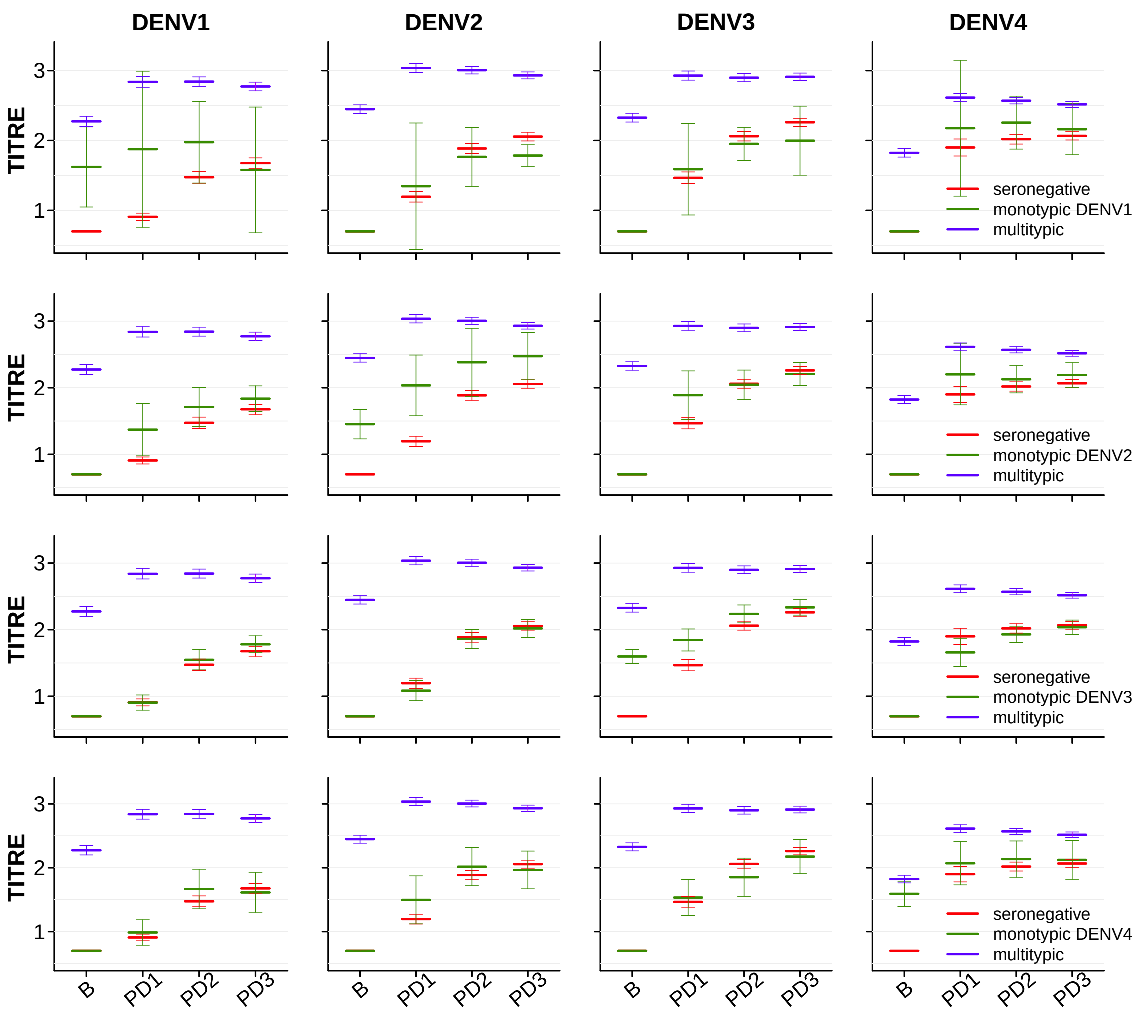

Figure S8: Mean and 95% CI of observed baseline (B), post-dose 1 (PD1), post-dose2 (PD2) and post-dose 3 (PD3) titres of seronegative, multitypic and monotypic DENV1 subjects (5/867) (row 1), monotypic DENV2 subjects (17/867) (row 2), monotypic DENV3 subjects (60/867) and monotypic DENV4 subjects (18/867). Titres are shown on a log10 scale.

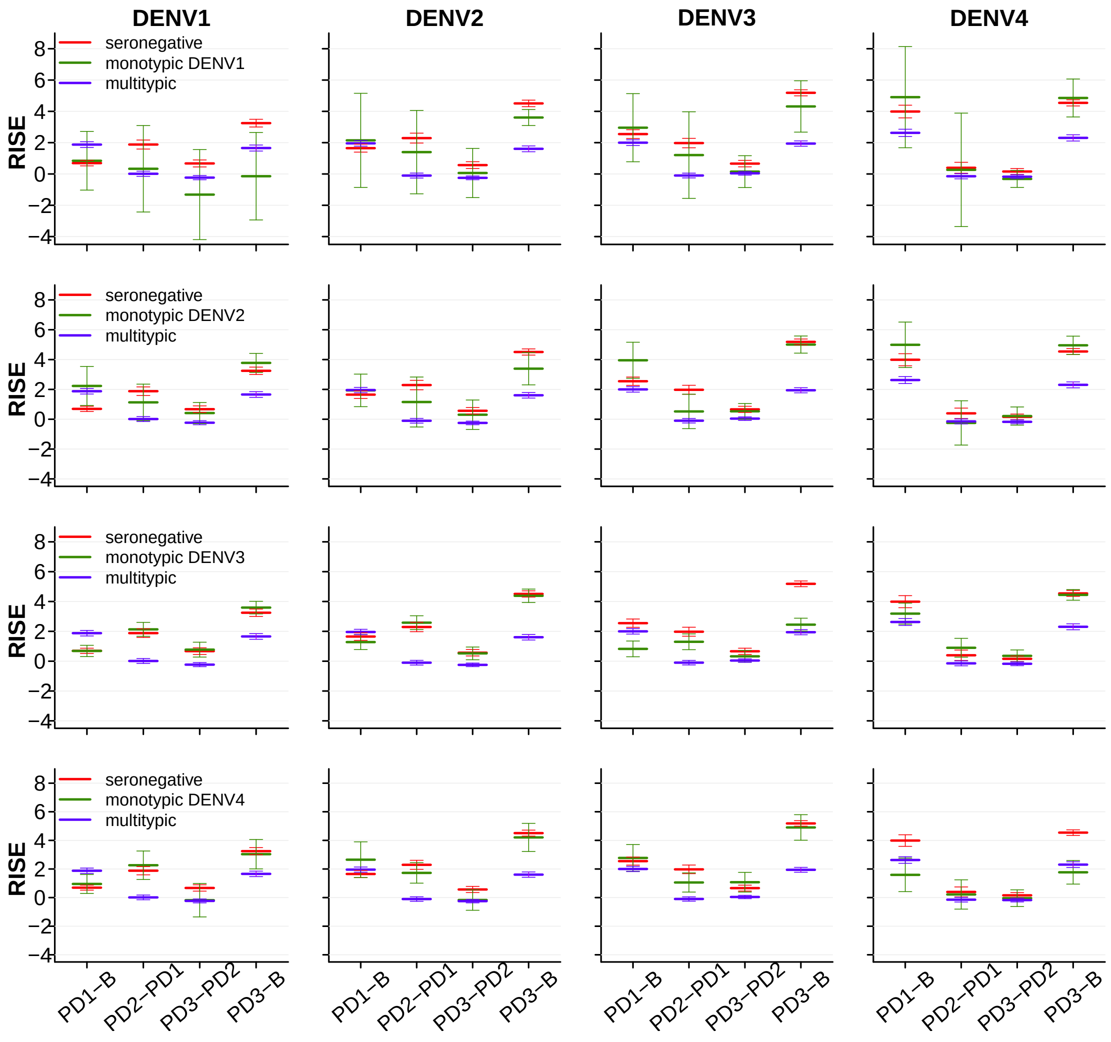

Figure S9: Mean and 95% CI of observed rises from baseline to post-dose 1 (PD1-B), from post-dose 1 to post-dose 2 (PD2-PD1), from post-dose2 to post-dose 3 (PD3-PD2) and from baseline to post-dose 3 (PD3-B) of seronegative, multitypic and monotypic DENV1 subjects (5/867) (row 1), monotypic DENV2 subjects (17/867) (row 2), monotypic DENV3 subjects (60/867) and monotypic DENV4 subjects (18/867). Increases in titres are shown on a log2 scale, according to the definition.

3.3.2 Definition 2 with threshold at 40

In this section we adopt the same definition given in section 3.3.1 but with threshold equal to 40 instead of 10. An antibody pattern is classified as seronegative if it has titre < 40 to all 4 DENV serotypes, monotypic if it has titre ≥ 40 to one DENV serotype only and multitypic if it has titre ≥ 40 to more than one DENV serotypes. For consistency, we define a JEV titre < 40 to be seronegative (JEV-) and a JEV titre ≥ 40 to be seropositive (JEV+). Figure S10 shows the mean and 95% confidence interval (CI) of the observed DENV titres and raises in titres in time in DENV seronegative, monotypic and multitypic profiles, according to the definition given in this section. Figures S11 and S12 show the mean and 95% confidence interval (CI) of the observed DENV titres in time and of the observed rises in titres between successive vaccinations respectively, having classified the subjects as DENV seronegative, monotypic profile with detected DENV1 titre, monotypic profile with detected DENV2 titre, monotypic profile with detected DENV3 titre, monotypic

profile with detected DENV4 titre or multitypic infection profile, according to the definition given in this section. Figures S13 and S14 show the mean and 95% CI of the observed DENV titres in time and of the observed rises in titres between successive vaccinations stratified by baseline immunological status against DENV and continent where the trial was conducted (i.e. South East Asia (SEA) or Latin America (LA)) (row 1), by baseline immunological status against DENV and trial (row 2) and by baseline immunological status against DENV and JEV (only for South East Asian studies) (row 3).

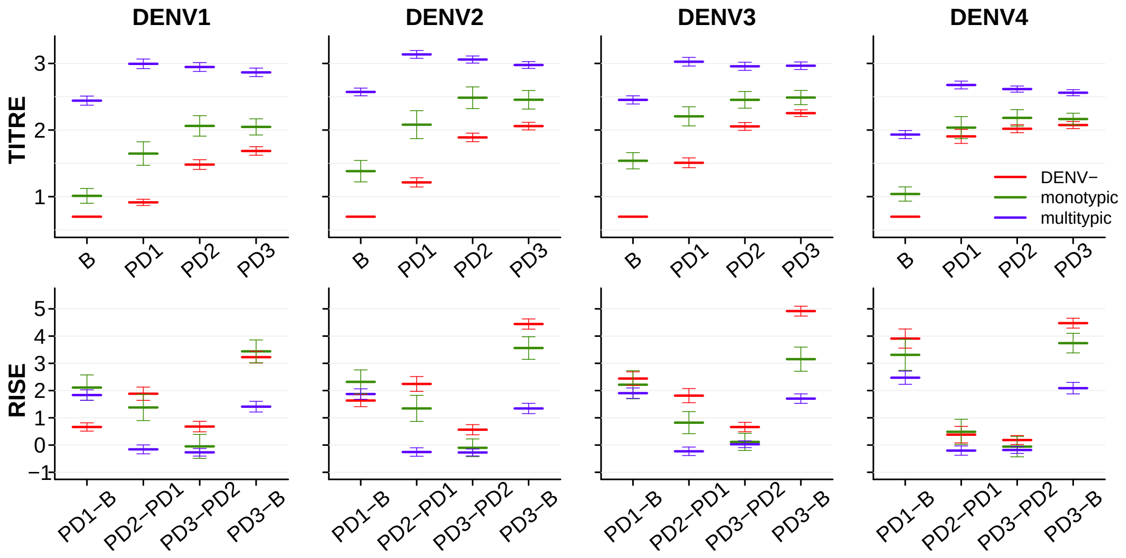

Figure S10: Mean and 95% CI of observed baseline (B), post-dose 1 (PD1), post-dose 2 (PD2) and post-dose 3 (PD3) titres (row 1) and rises in titres (row 2) for each DENV serotype (columns) by baseline immunological status against DENV (colour code). Here, DENV- denotes subjects with titre <40 for all DENV serotypes; monotypic denotes subjects with only one serotype titre $\geq$ 40; multitypic denotes subjects with titre $\geq$ 40 for at least two DENV serotypes. Undetectable titres (i.e. titres <10) are assigned a titre value of 5. Titres are shown on a log10 scale. Increases in titres are shown on a log2 scale, according to the definition.

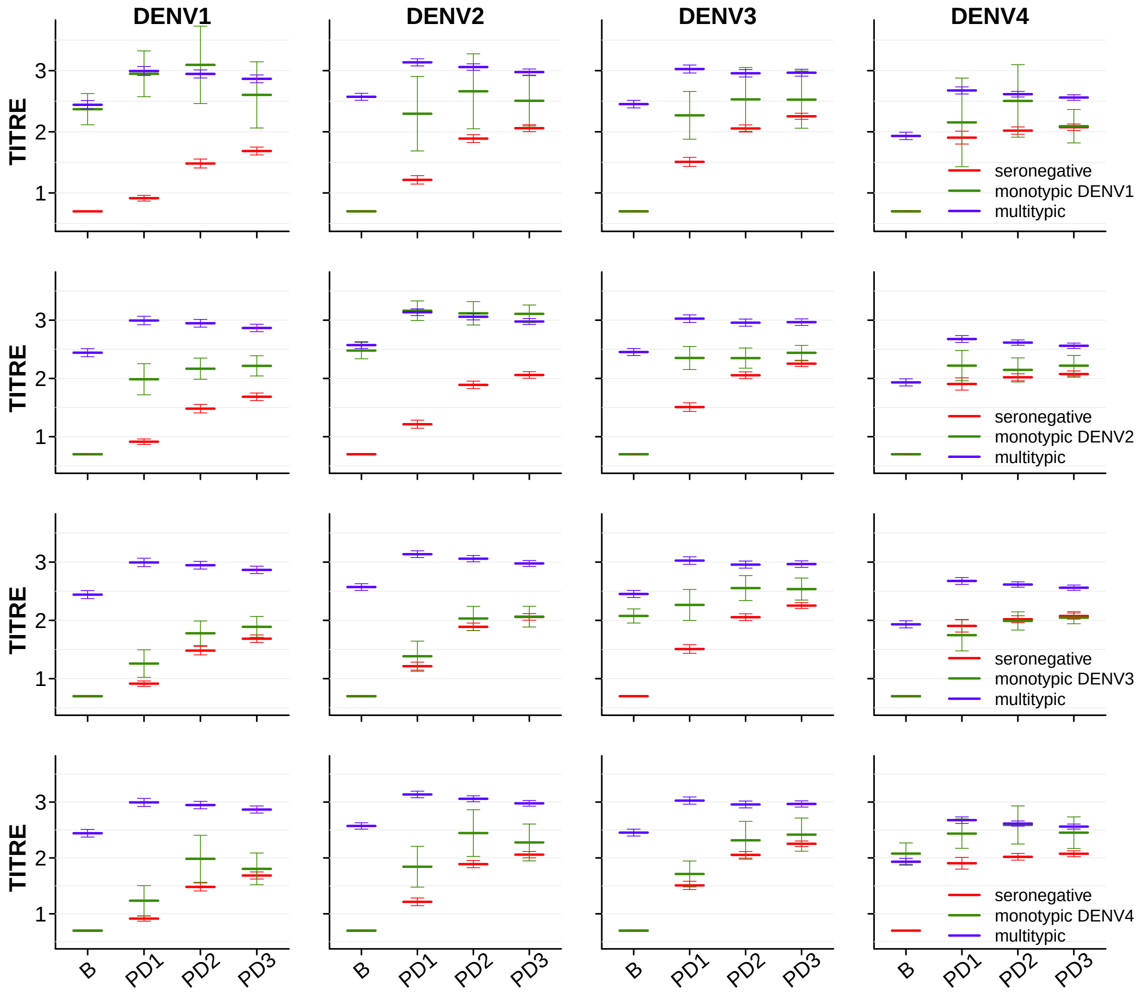

Figure S11: Mean and 95% CI of observed baseline (B), post-dose 1 (PD1), post-dose2 (PD2) and post-dose 3 (PD3) titres of seronegative, multitypic and monotypic DENV1 subjects (10/867) (row 1), monotypic DENV2 subjects (29/867) (row 2), monotypic DENV3 subjects (43/867) and monotypic DENV4 subjects (15/867). Here, seronegative denotes subjects with titre <40 for all DENV serotypes; monotypic denotes subjects with only one serotype titre $\geq$ 40; multitypic denotes subjects with titre $\geq$ 40 for at least two DENV serotypes. Undetectable titres (i.e. titres <10) are assigned a titre value of 5. Titres are shown on a log10 scale.

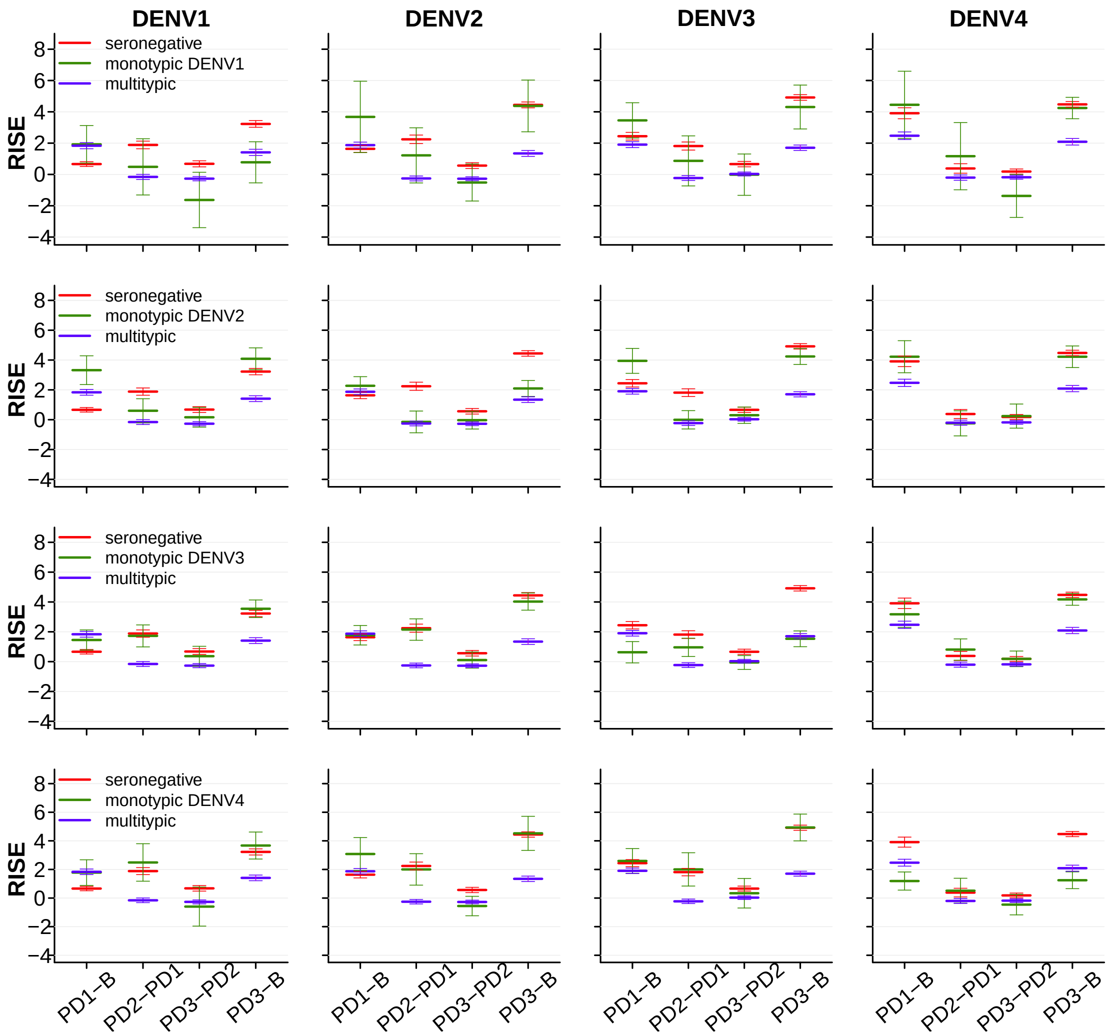

Figure S12: Mean and 95% CI of observed rises from baseline to post-dose 1 (PD1-B), from post-dose 1 to post-dose 2 (PD2-PD1), from post-dose2 to post-dose 3 (PD3-PD2) and from baseline to post-dose 3 (PD3-B) of seronegative, multitypic and monotypic DENV1 subjects (10/867) (row 1), monotypic DENV2 subjects (29/867) (row 2), monotypic DENV3 subjects (43/867) and monotypic DENV4 subjects (15/867). Here, seronegative denotes subjects with titre <40 for all DENV serotypes; monotypic denotes subjects with only one serotype titre $\geq$ 40; multitypic denotes subjects with titre $\geq$ 40 for at least two DENV serotypes. Undetectable titres (i.e. titres <10) are assigned a titre value of 5. Increases in titres are shown on a log2 scale, according to the definition of rise in titre.

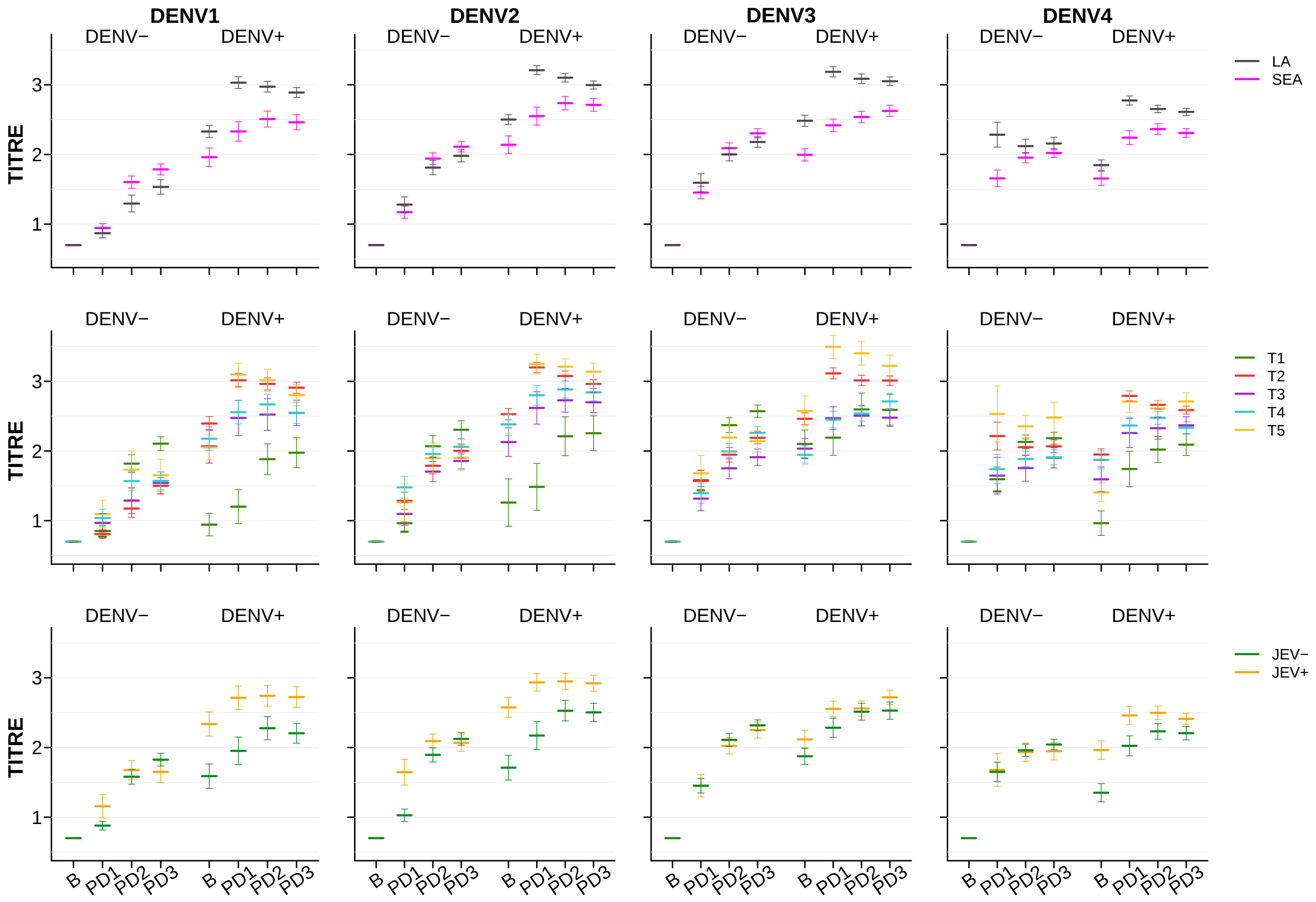

Figure S13: Mean and 95% CI of observed baseline (B), post-dose 1 (PD1), post-dose2 (PD2) and post-dose 3 (PD3) titres by baseline DENV immunological status and continent (LA=Latin America, SEA = South East Asia) (row 1), baseline DENV immunological status and study (row 2) and baseline DENV immunological status and baseline JEV immunological status (row 3). Here, DENV- denotes subjects with titre <40 for all DENV serotypes; DENV+ denotes subjects with titre ≥ 40 for at least one DENV serotype; JEV- denotes subjects with titre <40 for JEV; JEV+ denotes subjects with titre ≥ 40 for JEV. Undetectable titres (i.e. titres <10) are assigned a titre value of 5. Titres are shown in a log10 scale.

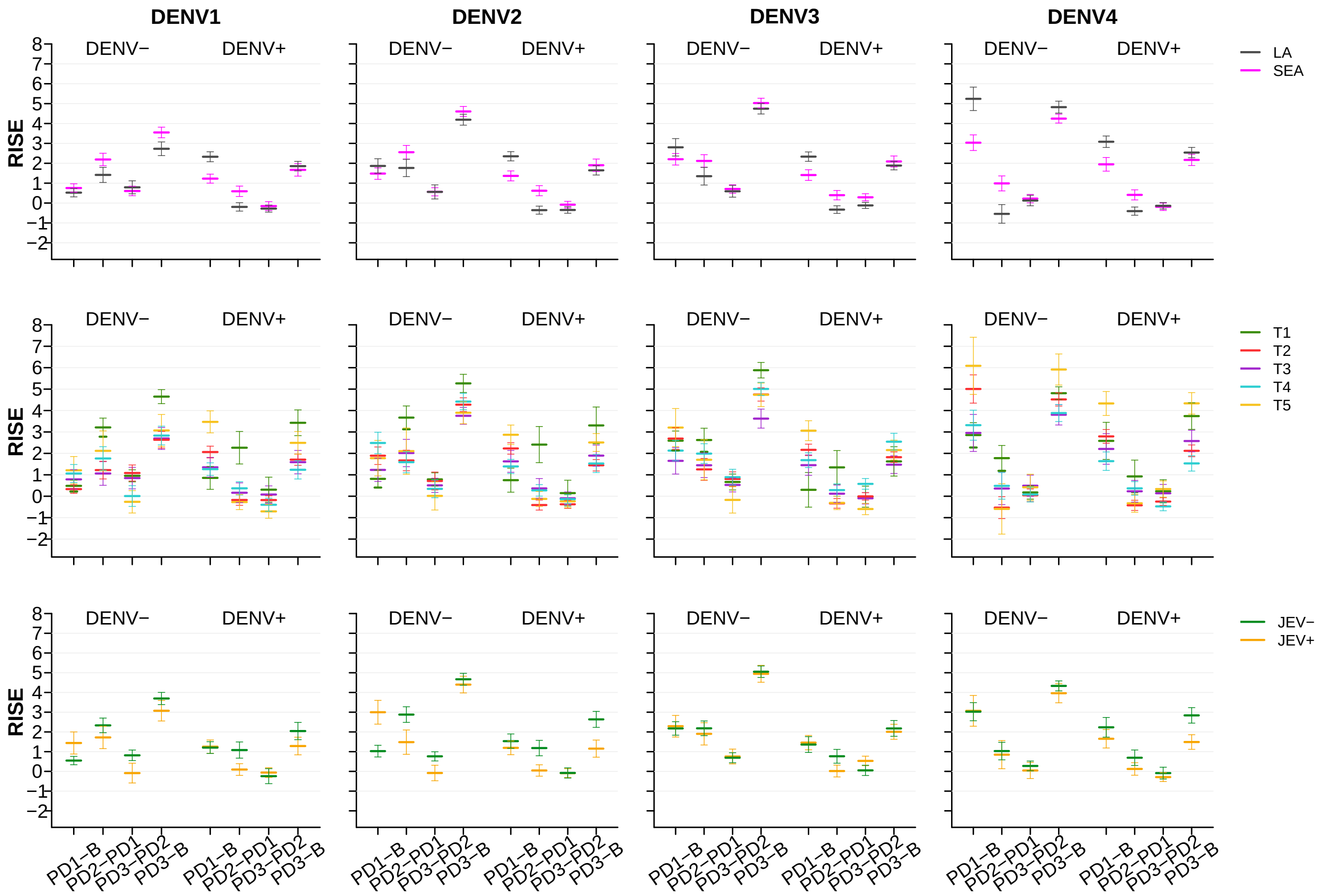

Figure S14: Mean and 95% CI of observed rises in tires between baseline and post-dose 1 (B-PD1), post-dose 1 and post-dose2 (PD1-PD2), post-dose 2 and post-dose 3 (PD2-PD3) and baseline and post-dose 3 (B-PD3) by baseline DENV immunological status and continent (LA=Latin America, SEA = South East Asia) (row 1), baseline DENV immunological status and study (row 2) and baseline DENV immunological status and baseline JEV immunological status (row 3). Here, DENV- denotes subjects with titre <40 for all DENV serotypes; DENV+ denotes subjects with titre $\geq$ 40 for at least one DENV serotype; JEV- denotes subjects with titre <40 for JEV; JEV+ denotes subjects with titre $\geq$ 40 for JEV. Undetectable titres (i.e. titres <10) are assigned a titre value of 5. Increases in titres are shown on a log2 scale, according to the definition of rise in titre.

In terms of multivariate analysis, we explored the effect of using the threshold of 40 to define undetectable (< 40) and detectable (≥ 40) titres. Under this assumption, the results obtained with models 1-11 are given in Table S15 and show that model 8 has the lowest AIC.

Models 8a-8g build on Model 8 and include a serotype-specific intercept for the gender, the age of the subject, the continent where the subject was enrolled, the baseline immunological DENV status of the subjects, the JEV titre (if detected), the baseline immunological status against JEV and the trial, respectively. The equation of Model 8g, which includes serotype-specific intercepts for each trial, is

$$Y_{ji} = X_{ji}(1-U_{ji})\beta_{1i} + U_{ji}\beta_{2i} + \sum_{k=1}^{4} X_{jk}(1-U_{jk})\beta_{3k} + \sum_{k=1}^{4} U_{jk}\beta_{4k} + T1_j\beta_{5i} + T2_j\beta_{6i} + + T3_j\beta_{7i} + T4_j\beta_{8i} + T5_j\beta_{9i} + \sum_{k\neq i} X_{ji}X_{jk}(1-U_{ji})(1-U_{jk})\gamma_{ki} + \sum_{k\neq i} U_{ji}U_{jk}\delta_{ki} + e_{ji} \quad (25)$$

Models 8h-8l extend Model 8g and add a serotype-specific intercept for the gender, the age of the subject, the baseline immunological DENV status of the subjects, the JEV titre (if detected) and the baseline immunological status against JEV, respectively.

Table S16 shows the results, in terms of goodness of fit, obtained with model variants 8a-8l. According to the AIC, model 8g is the best model overall. Tables S17 and S18 show the estimates and the correlation between the PD3 titres against the four DENV serotypes obtained using model 8g, respectively. Figure S15 shows the observed and expected mean and 5-95 percentiles of the PD3 titres versus the homologous baseline titres obtained with the best model (Model 8g), having simulated 100 independent realisations of the error by drawing from the multivariate normal distribution with zero mean and the estimated variance-covariance matrix Σ given in Table S17. Figure S16 shows the scatterplot of the observed and expected PD3 titres versus the homologous baseline titres having simulated a single random realisation of the error.

Model	Covariates	AIC	ΔAIC	Log-like	np	R_1^2	R_2^2	R_3^2	R_4^2
1	X_{ji}	4493.54	183.87	-2224.77	22	0.50	0.45	0.42	0.18
2	X_{ji}, $\max_{k\neq i}(X_{jk})$	4434.30	124.64	-2187.15	30	0.53	0.46	0.44	0.20
3	X_{ji}, $\text{mean}_{k\neq i}(X_{jk})$	4448.35	138.68	-2194.17	30	0.51	0.46	0.44	0.21
4	$X_{j1}, \ldots, X_{j4}$	4387.08	77.42	-2163.54	30	0.54	0.49	0.46	0.23
5	$X_{j1}, \ldots, X_{j4}$	4362.83	53.16	-2135.42	46	0.55	0.49	0.46	0.24
6	X_{ji}, $\max_{k\neq i}(X_{jk})$, $X_{ji}\max_{k\neq i}(X_{jk})$	4362.16	52.49	-2143.08	38	0.55	0.48	0.45	0.23
7	X_{ji}, $\text{mean}_{k\neq i}(X_{jk})$, $X_{ji}\text{mean}_{k\neq i}(X_{jk})$	4409.08	99.41	-2166.54	38	0.53	0.47	0.44	0.24
8	$X_{j1}, \ldots, X_{j4}$, $X_{ji}X_{jk}$ for $i \neq k$	**4309.67**	0	-2100.83	54	0.57	0.50	0.46	0.25
9	$X_{j1}, \ldots, X_{j4}$, $X_{ji}\max_{k\neq i}(X_{jk})$	4341.43	31.76	-2132.71	38	0.55	0.49	0.45	0.25
10	$X_{j1}, \ldots, X_{j4}$, $X_{ji}X_{jk}$ for $i \neq k$	4323.82	14.15	-2091.91	70	0.57	0.50	0.46	0.26
11	$X_{j1}, \ldots, X_{j4}$, $X_{ji}\max_{k\neq i}(X_{jk})$	4342.59	32.92	-2121.29	50	0.56	0.50	0.46	0.24

Table S15: Akaike's Information Criterion score (AIC), AIC difference from best model (ΔAIC), log-likelihood (Log-like), number of parameters estimated in the regression equation (np) and fraction of serotype-specific explained variance compared to the null model ($R_1^2, \ldots, R_4^2$) obtained by fitting the observed post dose 3 titres to the specified covariates, using the regression models in equations (1)-(11). The number of estimated parameters (np) includes the coefficients of the regression models β_{ki} ($k = 0, \ldots, p$) and the variance-covariances of the serotypes Σ_{il} ($i, l = 1, \ldots, 4$). The AIC of the best model is in bold.

Model	Additional covariate(s)	AIC	ΔAIC	Log-like	np	R_1^2	R_2^2	R_3^2	R_4^2
8a	F_j	4312.59	167.60	-2098.29	58	0.57	0.50	0.46	0.26
8b	A_j	4280.67	135.69	-2082.34	58	0.57	0.51	0.47	0.26
8c	L_j	4282.82	137.84	-2083.41	58	0.57	0.50	0.46	0.28
8d	P_j	4316.05	171.07	-2100.02	58	0.57	0.50	0.46	0.26
8e	JET_j	4282.74	137.76	-2079.37	62	0.57	0.50	0.46	0.28
8f	JE_{N_j}, JE_{P_j}	4287.13	142.15	-2081.57	62	0.57	0.50	0.46	0.28
8g	Ts_j ($s = 1, \ldots, 5$)	**4144.98**	0	-2002.49	70	0.59	0.51	0.50	0.32
8g	Ts_j	4144.98	0	-2002.49	70	0.59	0.51	0.50	0.32
8h	Ts_j , F_j	4145.83	0.85	-1998.92	74	0.59	0.51	0.51	0.32
8i	Ts_j , A_j	4150.35	5.36	-2001.17	74	0.59	0.51	0.50	0.32
8j	Ts_j , P_j	4151.52	6.54	-2001.76	74	0.59	0.51	0.50	0.32
8k	Ts_j , JET_j	4149.12	4.14	-1996.56	78	0.59	0.51	0.51	0.32
8l	Ts_j , JE_{N_j}, JE_{P_j}	4152.59	7.61	-1994.30	82	0.59	0.51	0.51	0.32

Table S16: Akaike's Information Criterion score (AIC), AIC difference from best model (ΔAIC), log-likelihood (Log-like), number of estimated parameters (np) and fraction of serotype-specific explained variance compared to the null model ($R_1^2, \ldots, R_4^2$) obtained by fitting the observed post dose 3 titres to the baseline titres as in model 8 plus the specified covariates. Models 8a-8g and 8h-8l have respectively 1 and 2 additional covariates compared to model 8. The AIC of the best model in each section of the table is in bold and the AIC differences are relative to the best model in each section. The number of estimated parameters (np) includes the coefficients of the regression models β_{ki} $(k = 0, \ldots, p)$ and the variance-covariances of the serotypes Σ_{il} $(i, l = 1, \ldots, 4)$.

Coefficient	Serotype i=1			Serotype i=2			Serotype i=3			Serotype i=4		
	MLE	SE	p-value	MLE	SE	p-value	MLE	SE	p-value	MLE	SE	p-value
β_{1i}	0.992	0.084	<0.001	0.622	0.073	<0.001	0.753	0.069	<0.001	1.173	0.109	<0.001
β_{2i}	1.259	0.123	<0.001	1.211	0.163	<0.001	1.189	0.126	<0.001	1.133	0.133	<0.001
β_{3i}	0.093	0.048	0.054	0.168	0.049	<0.001	0.144	0.048	0.003	-0.246	0.071	<0.001
β_{4i}	0.064	0.098	0.512	-0.191	0.103	0.064	-0.161	0.097	0.096	-0.486	0.120	<0.001
β_{5i}	1.182	0.250	<0.001	1.763	0.232	<0.001	1.593	0.224	<0.001	0.802	0.243	<0.001
β_{6i}	0.886	0.248	<0.001	1.673	0.230	<0.001	1.420	0.224	<0.001	0.792	0.243	0.001
β_{7i}	0.776	0.251	0.002	1.517	0.232	<0.001	1.108	0.225	<0.001	0.657	0.242	0.007
β_{8i}	0.765	0.251	0.002	1.643	0.231	<0.001	1.425	0.225	<0.001	0.599	0.245	0.014
β_{9i}	0.961	0.250	<0.001	1.743	0.232	<0.001	1.411	0.229	<0.001	1.101	0.246	<0.001
γ_{1i}	-	-	-	-0.092	0.019	<0.001	-0.057	0.020	0.004	0.001	0.019	0.960
γ_{2i}	-0.024	0.016	0.129	-	-	-	-0.083	0.018	<0.001	-0.019	0.019	0.321
γ_{3i}	-0.029	0.017	0.092	-0.079	0.018	<0.001	-	-	-	-0.019	0.019	0.310
γ_{4i}	-0.048	0.020	0.015	-0.098	0.023	<0.001	-0.069	0.022	0.002	-	-	-
δ_{1i}	-	-	-	0.195	0.112	0.083	0.251	0.108	0.021	-0.159	0.113	0.160
δ_{2i}	-0.300	0.133	0.024	-	-	-	0.328	0.112	0.003	-0.087	0.124	0.483
δ_{3i}	0.017	0.115	0.881	0.489	0.113	<0.001	-	-	-	-0.081	0.116	0.487
δ_{4i}	0.098	0.107	0.358	0.518	0.113	<0.001	0.334	0.107	0.002	-	-	-
Σ_{1i}	0.287	21.529	<0.001	0.139	14.249	<0.001	0.139	12.411	<0.001	0.092	10.071	<0.001
Σ_{2i}	0.139	14.249	<0.001	0.241	29.823	<0.001	0.112	12.102	<0.001	0.092	11.110	<0.001
Σ_{3i}	0.139	12.411	<0.001	0.112	12.102	<0.001	0.200	25.943	<0.001	0.075	10.917	<0.001
Σ_{4i}	0.092	10.071	<0.001	0.092	11.110	<0.001	0.075	10.917	<0.001	0.192	21.529	<0.001

Table S17: Maximum likelihood estimates (MLE), standard errors (SE) and two-sided p-values of the coefficients of model variant 8g. The equation of model variant 8g is $Y_{ji} = X_{ji}(1-U_{ji})\beta_{1i} + U_{ji}\beta_{2i} + \sum_{k=1}^{4} X_{jk}(1-U_{jk})\beta_{3k} + \sum_{k=1}^{4} U_{jk}\beta_{4k} + T1_j\beta_{5i} + T2_j\beta_{6i} + T3_j\beta_{7i} + T4_j\beta_{8i} + T5_j\beta_{9i} + \sum_{k\neq i} X_{ji}X_{jk}(1-U_{ji})(1-U_{jk})\gamma_{ki} + \sum_{k\neq i} U_{ji}U_{jk}\delta_{ki} + e_{ji}$.

ρ_{il}	l=1	l=2	l=3	l=4
$i=1$	1	0.53	0.58	0.39
$i=2$	0.53	1	0.51	0.43
$i=3$	0.58	0.51	1	0.38
$i=4$	0.39	0.43	0.38	1

Table S18: Estimated correlations between the PD3 titres against DENV1-4 using model variant 8g.

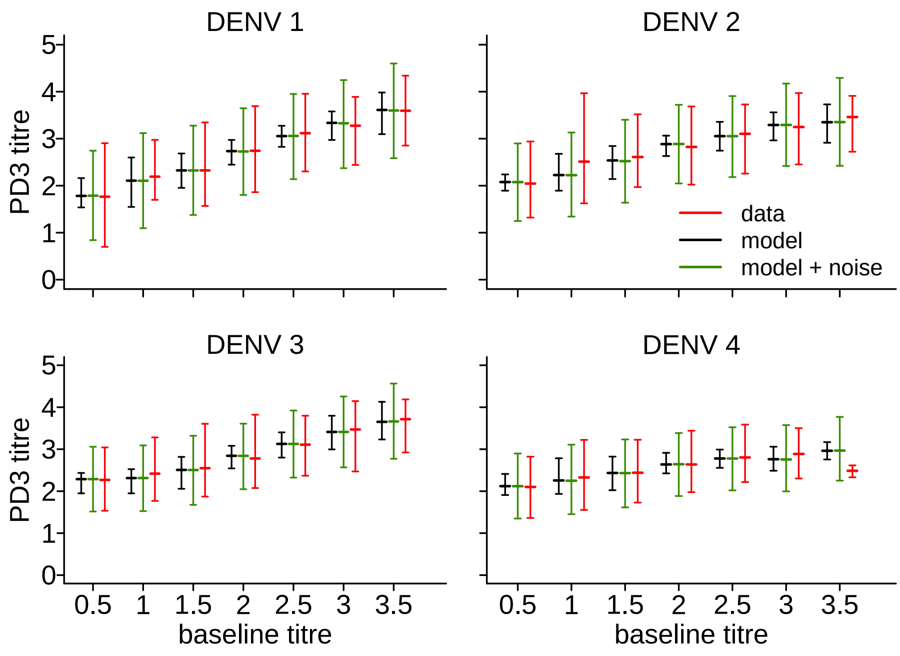

Figure S15: Observed (red), expected (black) and expected with simulated noise (green) mean and 5-95 percentiles of the post-dose 3 (PD3) titres versus the homologous baseline titres, obtained with the best model (model 8g). Titres are shown on a log10 scale and baseline titres are grouped in bins of width 0.5 (the first bin representing undetectable titres at baseline).

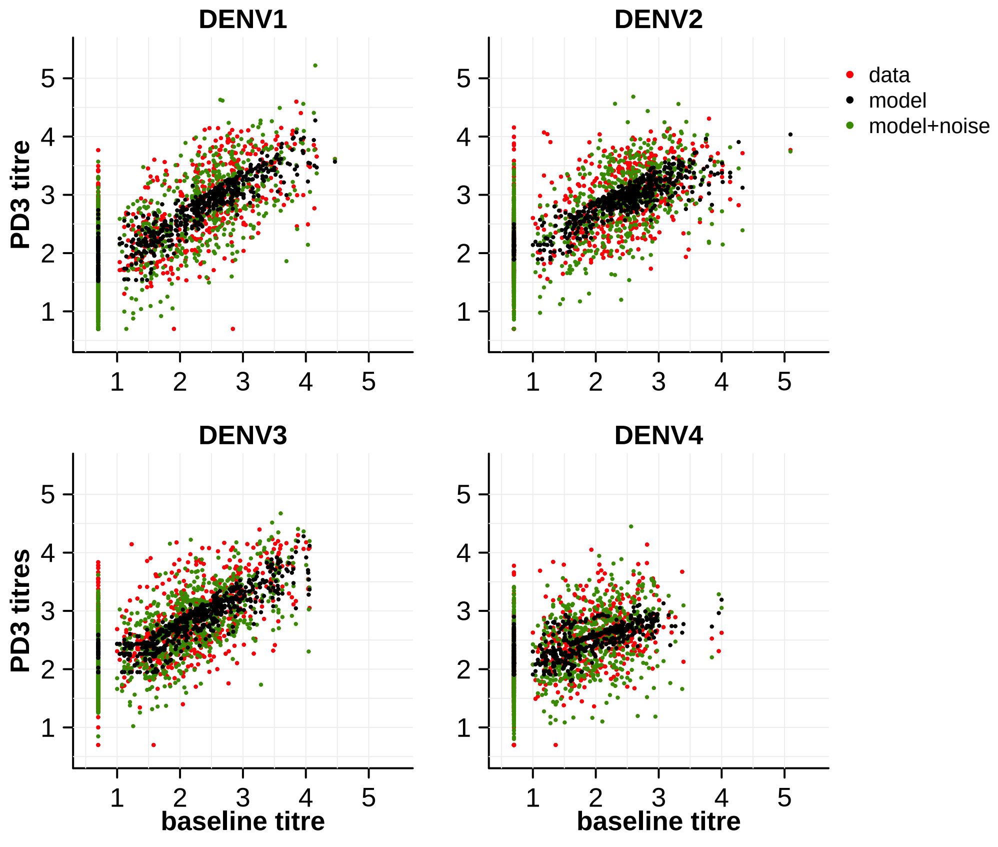

Figure S16: Observed (red), average expected (black) and expected with simulated noise (green) PD3 titres versus the observed homologous baseline titre, obtained with model 8g. The noise has been simulated by drawing a single realisation from a multivariate normal distribution with zero mean and the variance-covariance matrix Σ estimated with model 8g. Titres are shown on a log10 scale.

3.3.3 Definition 3 with threshold at 10

In this section we present the results of the descriptive analysis obtained using the following definitions: an antibody pattern is classified as seronegative if it has titre < 10 to all 4 DENV serotpyes; monotypic if it has titre ≥ 10 to one DENV serotype only or if it has titre ≥ 10 to more than one DENV serotypes with ≥ 6 fold higher response of the dominant (i.e. highest) DENV serotype versus the second dominant DENV serotype; multitypic if it has titre ≥ 10 to more than one DENV serotypes with < 6 fold higher response of the dominant (i.e. highest) DENV serotype versus the second dominant DENV serotype. For monotypic subjects, the dominant DENV serotype is assumed to be the serotype of the previous DENV infection. Figure S17 shows the mean and 95% confidence interval (CI) of the observed DENV titres and raises in titres in time in DENV seronegative, monotypic and multitypic profiles, according to the definitions given in this section. Figures S18 and S19 show the mean and 95% confidence interval (CI) of the observed DENV titres in time and of the observed rises in titres between successive vaccinations respectively, having classified the subjects as DENV seronegative, monotypic profile with detected DENV1 titre, monotypic profile with detected DENV2 titre, monotypic profile with detected DENV3 titre, monotypic profile with detected DENV4 titre or multitypic infection profile, according to the definitions given in this section.

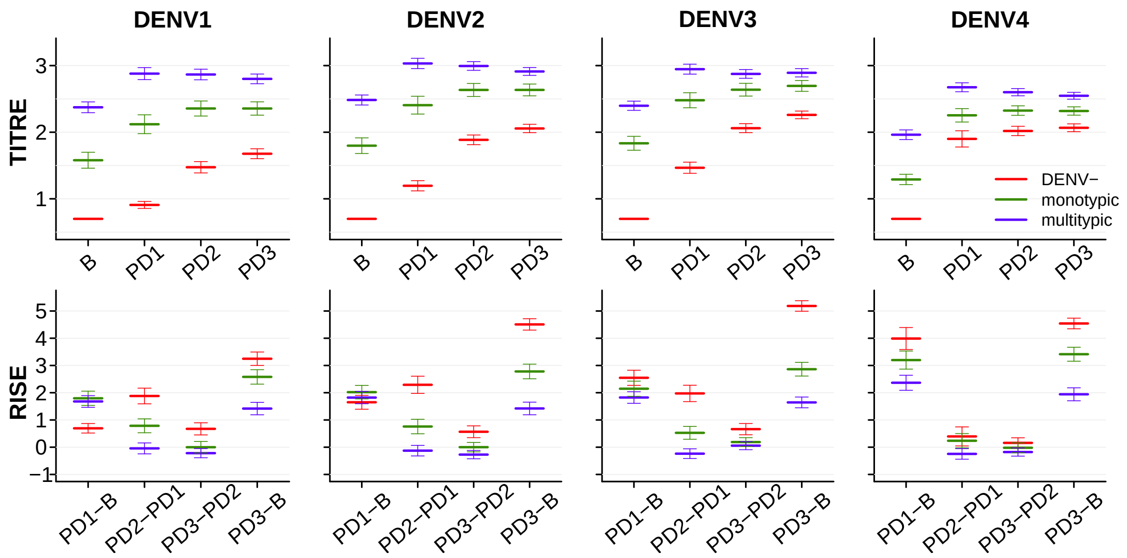

Figure S17: Mean and 95% CI of observed baseline (B), post-dose 1 (PD1), post-dose 2 (PD2) and post-dose 3 (PD3) titres (row 1) and rises in titres (row 2) for each DENV serotype (columns) by baseline immunological status against DENV (colour code). Here, DENV- denotes subjects with titre <10 for all DENV serotypes; monotypic denotes subjects with titre ≥ 10 to only one DENV serotype or with titre ≥ 10 to more than one DENV serotype and ≥ 6 higher response of the dominant titre versus the second highest titre; multitypic denotes subjects with titre ≥ 10 for at least two DENV serotypes and < 6 higher response of the dominant titre versus the second highest titre. Undetectable titres (i.e. titres <10) are assigned a titre value of 5. Titres are shown on a log10 scale. Increases in titres are shown on a log2 scale, according to the definition.

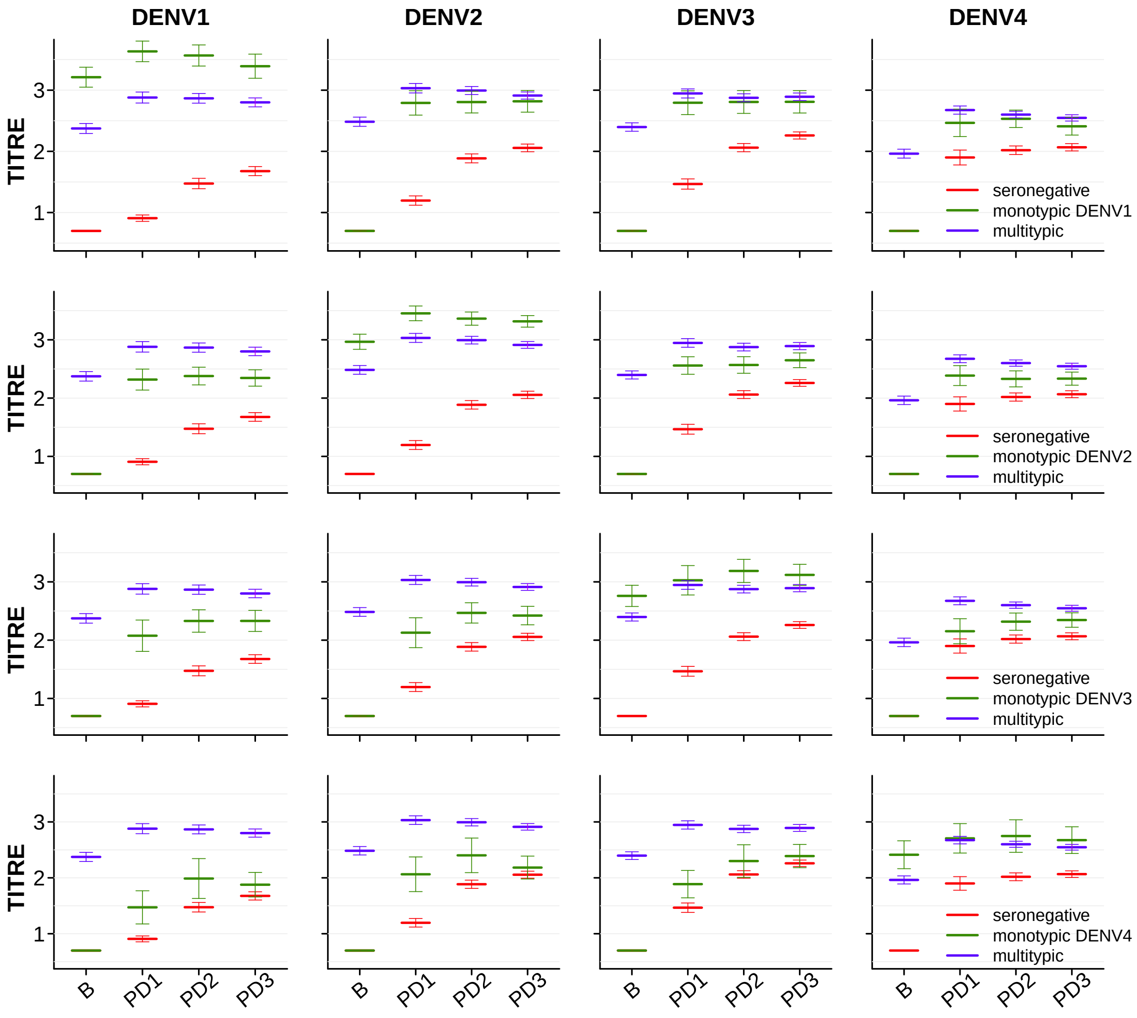

Figure S18: Mean and 95% CI of observed baseline (B), post-dose 1 (PD1), post-dose2 (PD2) and post-dose 3 (PD3) titres of seronegative, multitypic and monotypic DENV1 subjects (47/867) (row 1), monotypic DENV2 subjects (71/867) (row 2), monotypic DENV3 subjects (72/867) and monotypic DENV4 subjects (20/867). Titres are shown on a log10 scale.

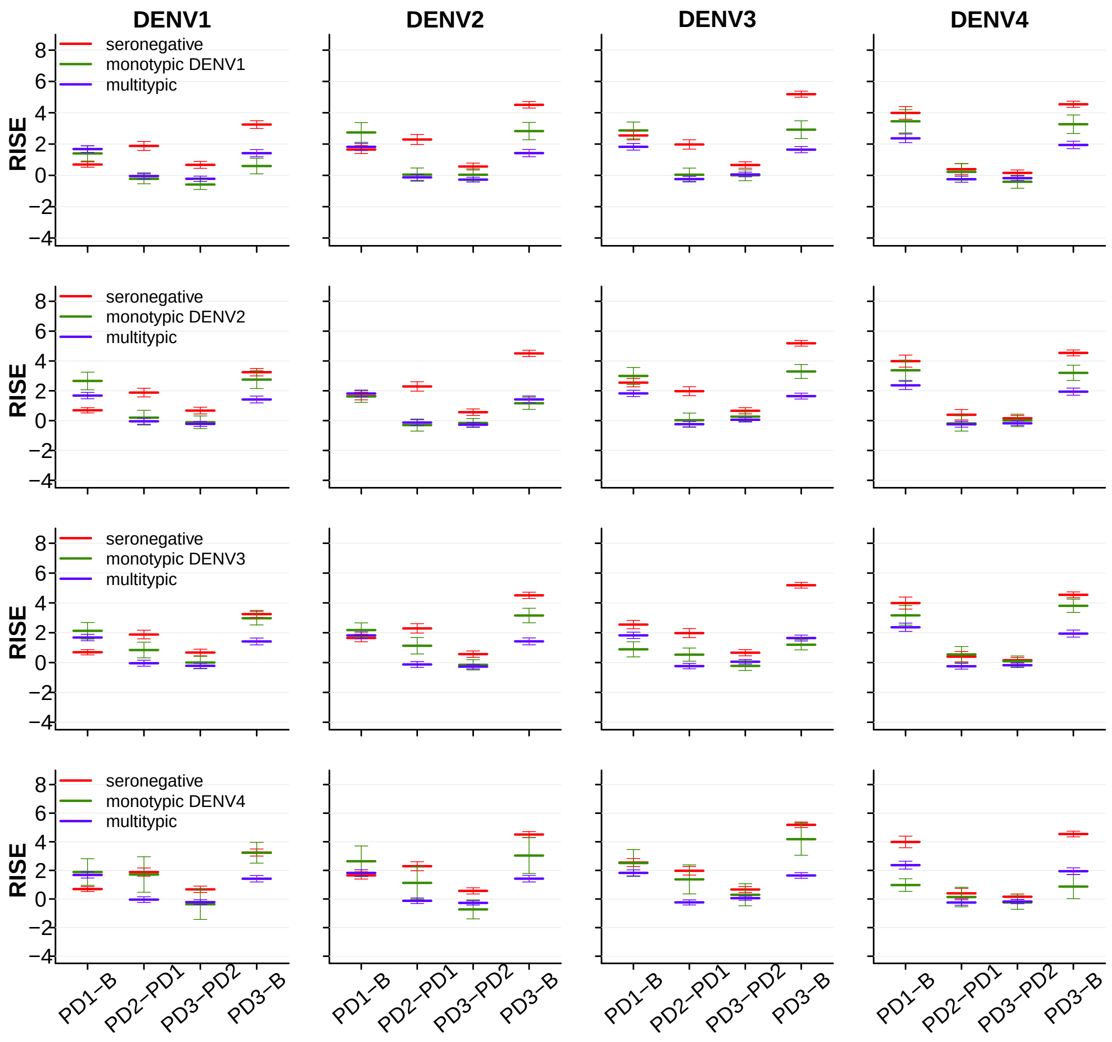

Figure S19: Mean and 95% CI of observed rises from baseline to post-dose 1 (PD1-B), from post-dose 1 to post-dose 2 (PD2-PD1), from post-dose2 to post-dose 3 (PD3-PD2) and from baseline to post-dose 3 (PD3-B) of seronegative, multitypic and monotypic DENV1 subjects (47/867) (row 1), monotypic DENV2 subjects (71/867) (row 2), monotypic DENV3 subjects (72/867) and monotypic DENV4 subjects (20/867). Increases in titres are shown on a log2 scale, according to the definition.

3.3.4 Definition 3 with threshold at 40

In this section we adopt the same definitions given in 3.3.3 but with threshold at 40 instead of 10. An antibody pattern is classified as seronegative if it has titre $<$ 40 to all 4 DENV serotpyes; monotypic if it has titre $\geq$ 40 to one DENV serotype only or if it has titre $\geq$ 40 to more than one DENV serotypes with $\geq$ 6 fold higher response of the dominant (i.e. highest) DENV serotype versus the second dominant DENV serotype; multitypic if it has titre $\geq$ 40 to more than one DENV serotypes with $<$ 6 fold higher response of the dominant (i.e. highest) DENV serotype versus the second dominant DENV serotype. For monotypic subjects, the dominant DENV serotype is assumed to be the serotype of the previous DENV infection. Figure S20 shows the mean and 95% confidence interval (CI) of the observed DENV titres and raises in titres in time in DENV seronegative, monotypic and multitypic profiles, according to the definitions given in this section. Figures S21 and S22 show the mean and 95% confidence interval (CI) of the observed

DENV titres in time and of the observed rises in titres between successive vaccinations respectively, having classified the subjects as DENV seronegative, monotypic profile with detected DENV1 titre, monotypic profile with detected DENV2 titre, monotypic profile with detected DENV3 titre, monotypic profile with detected DENV4 titre or multitypic infection profile, according to the definitions given in this section.

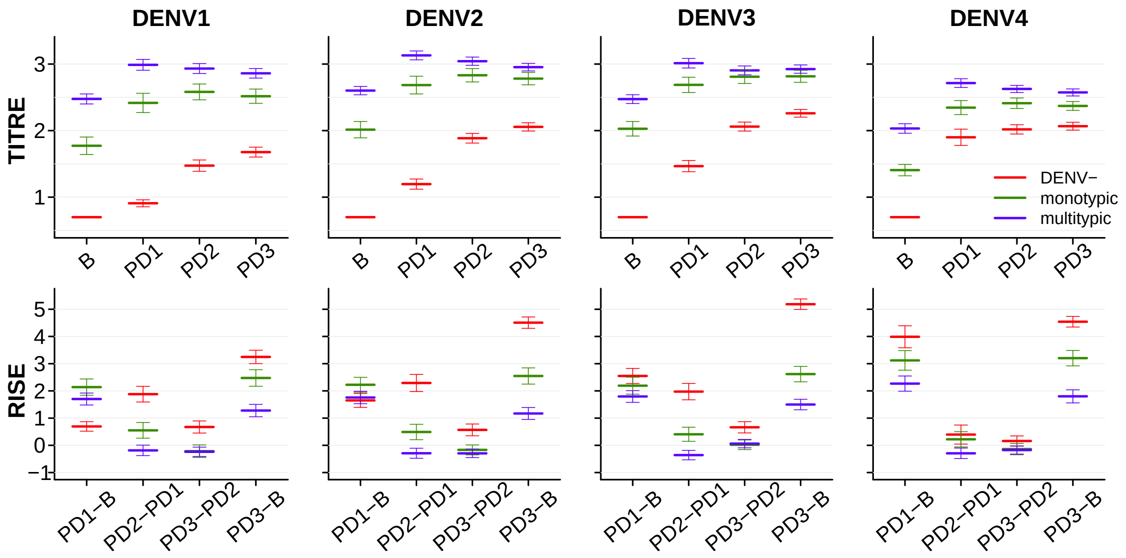

Figure S20: Mean and 95% CI of observed baseline (B), post-dose 1 (PD1), post-dose 2 (PD2) and post-dose 3 (PD3) titres (row 1) and rises in titres (row 2) for each DENV serotype (columns) by baseline immunological status against DENV (colour code). Here, DENV- denotes subjects with titre <40 for all DENV serotypes; monotypic denotes subjects with titre $\geq$ 40 to only one DENV serotype or with titre $\geq$ 40 to more than one DENV serotype and $\geq$ 6 higher response of the dominant titre versus the second highest titre; multitypic denotes subjects with titre $\geq$ 40 for at least two DENV serotypes and $<$ 6 higher response of the dominant titre versus the second highest titre. Undetectable titres (i.e. titres <10) are assigned a titre value of 5. Titres are shown on a log10 scale. Increases in titres are shown on a log2 scale, according to the definition.

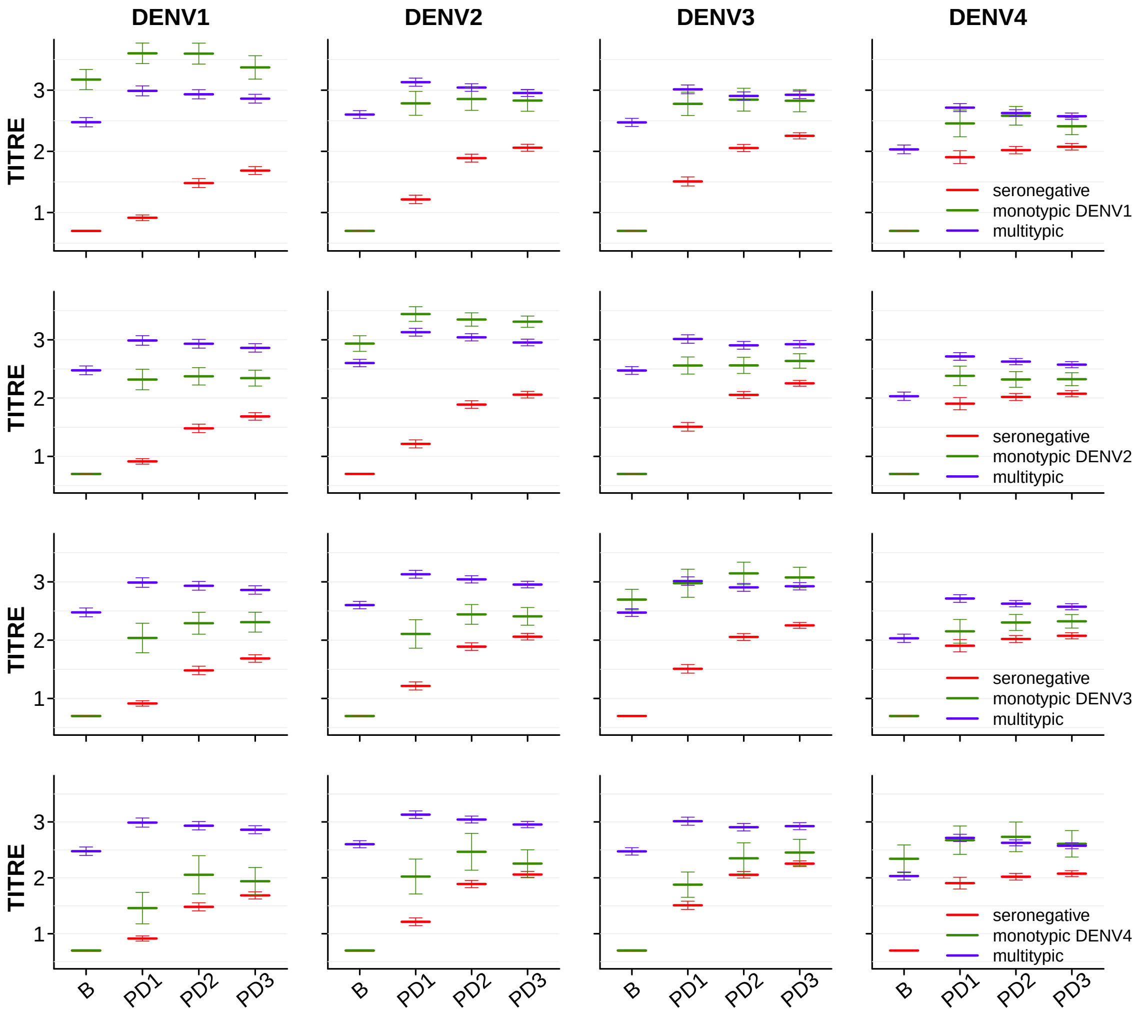

Figure S21: Mean and 95% CI of observed baseline (B), post-dose 1 (PD1), post-dose2 (PD2) and post-dose 3 (PD3) titres of seronegative, multitypic and monotypic DENV1 subjects (49/867) (row 1), monotypic DENV2 subjects (73/867) (row 2), monotypic DENV3 subjects (79/867) and monotypic DENV4 subjects (22/867). Titres are shown on a log10 scale.

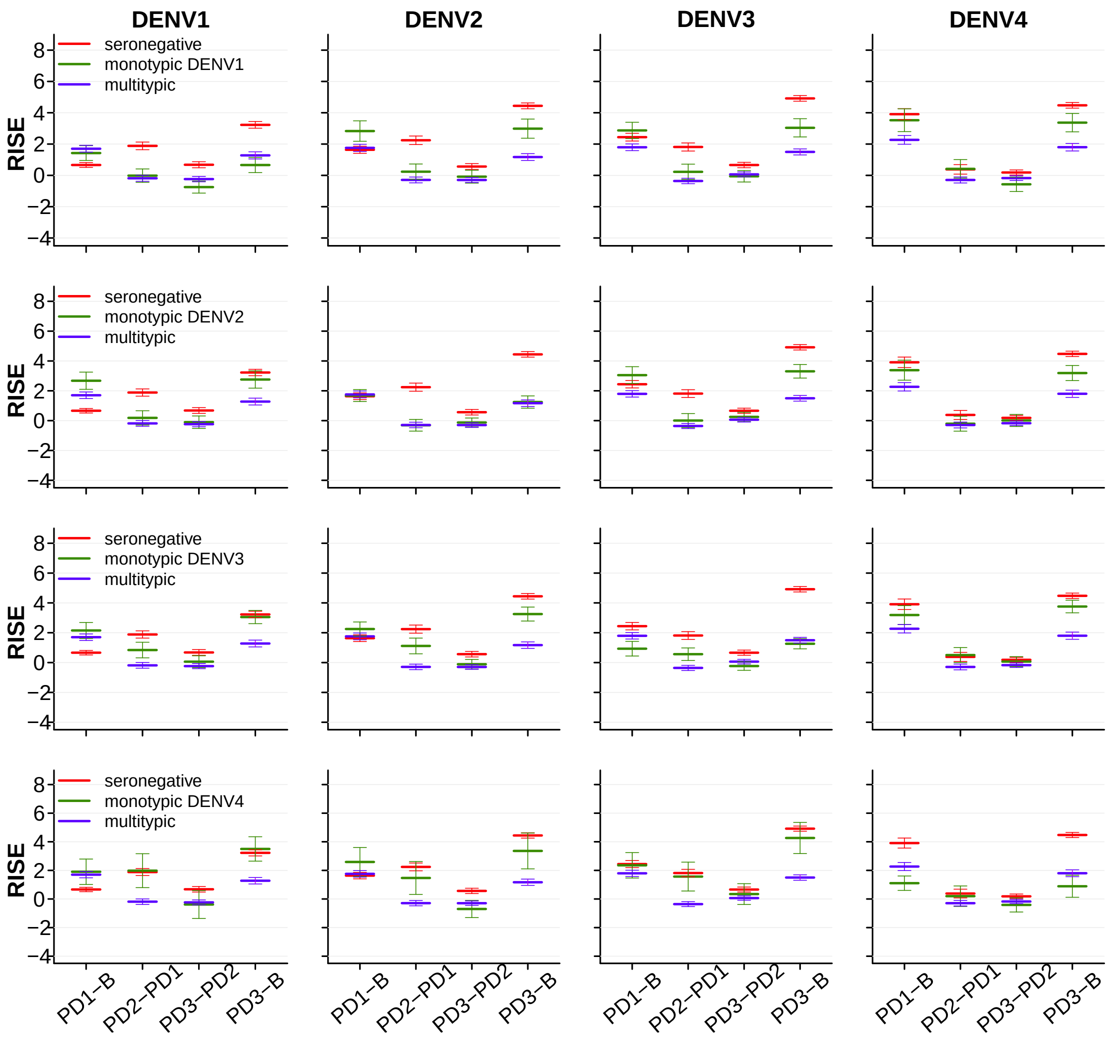

Figure S22: Mean and 95% CI of observed rises from baseline to post-dose 1 (PD1-B), from post-dose 1 to post-dose 2 (PD2-PD1), from post-dose2 to post-dose 3 (PD3-PD2) and from baseline to post-dose 3 (PD3-B) of seronegative, multitypic and monotypic DENV1 subjects (49/867) (row 1), monotypic DENV2 subjects (73/867) (row 2), monotypic DENV3 subjects (79/867) and monotypic DENV4 subjects (22/867). Increases in titres are shown on a log2 scale, according to the definition.

3.3.5 Comparison between definitions

Tables S19 and S20 summarise the baseline immunological characteristics of the subjects included in the descriptive analysis according to the definitions given in sections 3.3.1-3.3.4. The baseline immunological characteristics of the subjects included in the descriptive analysis according to definition 1 are given in Table 1 of the main text.

Study	Definition 2 (T=10)				Definition 2 (T=40)			
	DENV-	Mono	Multi	JEV+	DENV-	Mono	Multi	JEV+
T1	61 (53%)	39 (34%)	16 (14%)	6 (5%)	84 (72%)	24 (21%)	8 (7%)	1 (1%)
T2	91 (25%)	16 (4%)	254 (70%)	-	104 (29%)	25 (7%)	232 (64%)	-
T3	34 (30%)	19 (17%)	60 (53%)	42 (37%)	50 (44%)	16 (14%)	47 (42%)	31 (27%)
T4	55 (29%)	21(11%)	112 (60%)	151 (80%)	69 (37%)	22 (12%)	97 (52%)	121 (64%)
T5	26 (29%)	5 (6%)	58 (65%)	-	29 (33%)	10 (11%)	50 (56%)	-

Table S19: Baseline immunological characteristics of the subjects included in the descriptive analysis according to definition 2 with threshold at 10 and 40 given in sections 3.3.1 and 3.3.2, respectively.

Study	Definition 3 (T=10)				Definition 3 (T=40)			
	DENV-	Mono	Multi	JEV+	DENV-	Mono	Multi	JEV+
T1	61 (53%)	48 (41%)	7 (6%)	6 (5%)	84 (72%)	30 (36%)	2 (2%)	1 (1%)
T2	91 (25%)	81 (22%)	189 (52%)	-	104 (29%)	74 (20%)	183 (51%)	-
T3	34 (30%)	44 (39%)	35 (31%)	42 (37%)	50 (44%)	35 (31%)	28 (25%)	31 (27%)
T4	55 (29%)	66 (35%)	67 (36%)	151 (80%)	69 (37%)	56 (30%)	63 (34%)	121 (64%)
T5	26 (29%)	29 (33%)	34 (38%)	-	29 (33%)	28 (31%)	32 (36%)	-

Table S20: Baseline immunological characteristics of the subjects included in the descriptive analysis according to definition 3 with threshold at 10 and 40 given in sections 3.3.3 and 3.3.4, respectively.
